# A Large and Intact Viral Particle Penetrates the Endoplasmic Reticulum Membrane to Reach the Cytosol

**DOI:** 10.1371/journal.ppat.1002037

**Published:** 2011-05-12

**Authors:** Takamasa Inoue, Billy Tsai

**Affiliations:** Department of Cell and Developmental Biology, University of Michigan Medical School, Ann Arbor, Michigan, United States of America; Fred Hutchinson Cancer Research Center, United States of America

## Abstract

Non-enveloped viruses penetrate host membranes to infect cells. A cell-based assay was used to probe the endoplasmic reticulum (ER)-to-cytosol membrane transport of the non-enveloped SV40. We found that, upon ER arrival, SV40 is released into the lumen and undergoes sequential disulfide bond disruptions to reach the cytosol. However, despite these ER-dependent conformational changes, SV40 crosses the ER membrane as a large and intact particle consisting of the VP1 coat, the internal components VP2, VP3, and the genome. This large particle subsequently disassembles in the cytosol. Mutant virus and inhibitor studies demonstrate VP3 and likely the viral genome, as well as cellular proteasome, control ER-to-cytosol transport. Our results identify the sequence of events, as well as virus and host components, that regulate ER membrane penetration. They also suggest that the ER membrane supports passage of a large particle, potentially through either a sizeable protein-conducting channel or the lipid bilayer.

## Author Summary

Biological membranes represent a major barrier during viral infection. While the mechanism by which an enveloped virus breaches the limiting membrane of a host cell is well-characterized, this membrane penetration process is poorly understood for non-enveloped viruses. Indeed, most available insights on membrane transport of non-enveloped viruses are built upon in vitro studies. Here we established a cell-based assay to elucidate the molecular mechanism by which the non-enveloped SV40 penetrates the endoplasmic reticulum (ER) membrane to access the cytosol, a critical step in infection. Strikingly, we uncovered SV40 breaches the ER membrane as a large and intact viral particle, despite the conformational changes it experiences in the ER lumen. This result suggests that the ER membrane can accommodate translocation of a large protein complex, possibly through either a sizeable protein channel or the ER membrane bilayer. In addition to this finding, we also pinpoint viral and host components that control the ER-to-cytosol membrane transport event. Together, our data illuminate the cellular mechanism by which a non-enveloped virus penetrates the limiting membrane of a target cell during infection.

## Introduction

The mechanism by which non-enveloped viruses such as simian virus 40 (SV40) and the murine polyomavirus (mPy) penetrate the host cell's membrane to cause infection is enigmatic. However, a general model describing how they breach this membrane based largely on in vitro studies is emerging [1], [2]. In this model, the virus undergoes conformational changes by interacting with host factors, culminating in the formation of a hydrophobic viral particle or release of a lytic peptide. They then engage the limiting membrane to disrupt its integrity, enabling the virus to cross the membrane. As it is unknown whether this scenario reflects the pathway in cells, establishing a cell-based assay that monitors non-enveloped virus membrane penetration affords the opportunity to study this event's physiological mechanism. Important questions include: what reaction sequence initiates membrane penetration? What is the nature of the viral conformational change and identity of the membrane penetrating species? What viral and host components control the penetration process, and how is membrane transport achieved?

Here we address SV40's membrane transport process. Structurally, SV40 is composed of 72 pentamers of the VP1 coat assembled into an icosahedral viral capsid [3], [4]. Each VP1 pentamer engages the internal proteins VP2 and VP3 through hydrophobic interactions [5]. VP1 also binds to the ∼5 kb viral DNA genome buried within the virus through electrostatic interactions. Three additional forces support the overall viral architecture. First, disulfide bonds present throughout the virus stabilize it [4]. Second, the VP1 C-terminus invades a neighboring VP1 pentamer to provide inter-pentamer support [3]. And third, calciums bound to the virus clamp together different pentamers to increase capsid stabilization [4].

To infect cells, SV40 VP1 binds to the glycolipid ganglioside GM1 on the host cell surface [6], inducing membrane tubulation that initiates internalization [7]. The virus-receptor complex is then transported to the pH neutral caveosomes [8] or the low pH endolysosomes [9]. Regardless of the pathway, the virus subsequently sorts to the endoplasmic reticulum (ER). Upon arrival of the virus-receptor complex to the ER [10], SV40 is proposed to disassemble to cross the ER membrane and reach the cytosol [11]. From the cytosol, a subviral core particle transports into the nucleus where transcription and replication of the viral DNA ensue, leading to lytic infection or cell transformation.

Reactions controlling SV40's ER-to-cytosol transport, a decisive infection event, are not fully understood. How do ER-initiated events propel the virus to the cytosol? What is the identity of the membrane penetrating species? What viral, ER, and cytosolic components regulate this process? While a report suggests that the ER associated degradation (ERAD) machinery mediates SV40 infection [12], how this machinery geared normally to handle endogenous proteins much smaller than SV40 (∼50 nm in diameter) promotes membrane transport of the larger viral particle is unclear.

Here we established a cell-based assay to elucidate SV40's ER-to-cytosol membrane penetration. Our data demonstrate that, upon ER arrival, SV40 is released into the ER lumen and undergoes sequential disulfide bond modification as it moves to the cytosol. Despite these reactions, a large and intact SV40 intermediate penetrates the ER membrane to reach the cytosol where it disassembles. We also pinpoint viral and host components that regulate the penetration process. This assay thus provides the opportunity to illuminate SV40's membrane penetration mechanism in a cellular setting.

## Results

### Establishment of a cell based ER-to-cytosol membrane penetration assay for SV40

**Figure 1:**
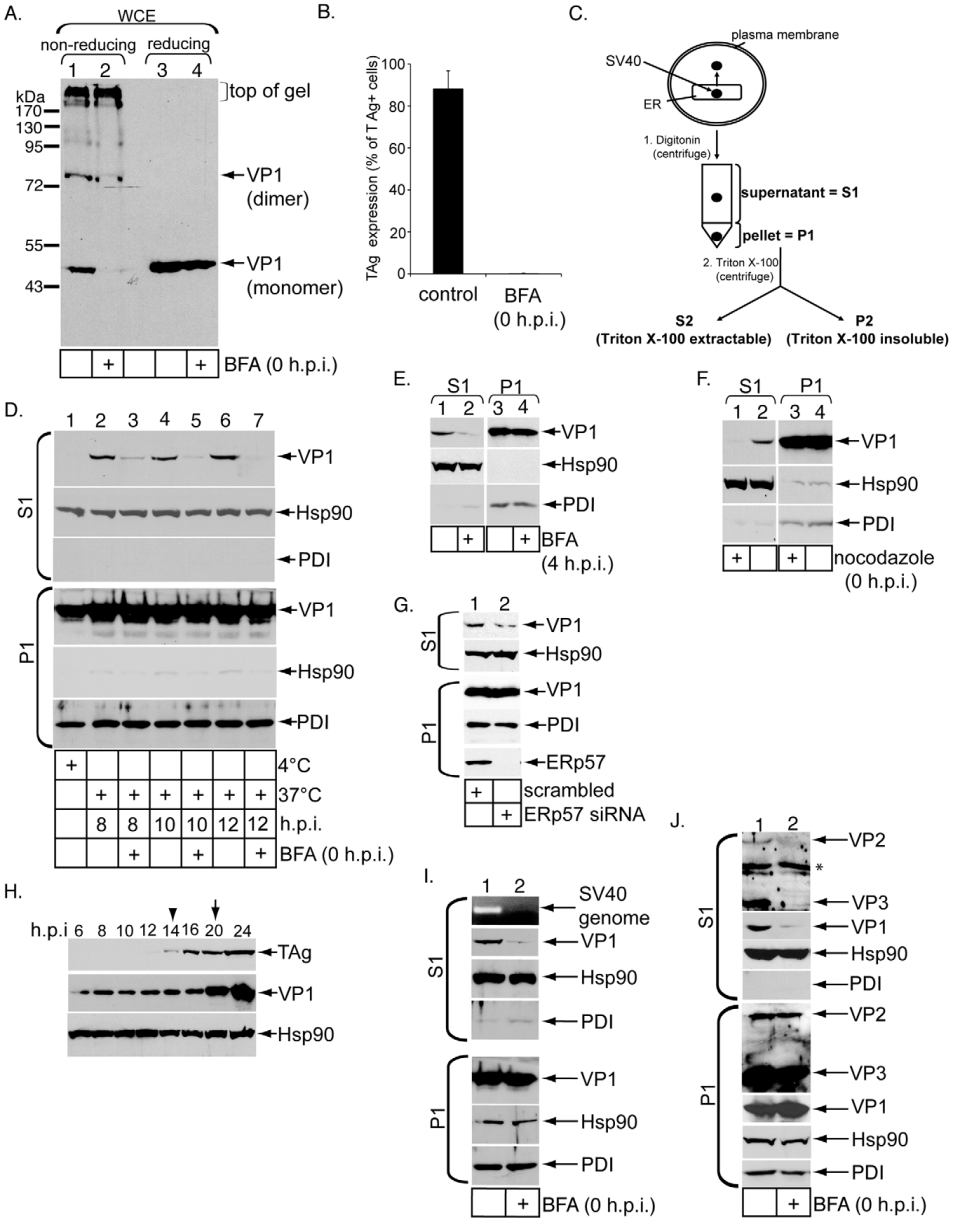
Establishment of a cell-based ER-to-cytosol membrane penetration assay for SV40. (A) SV40-infected cells were treated with or without BFA at infection (0 h.p.i.), and infection allowed for 12 hrs. WCE was prepared and analyzed by non-reducing and reducing SDS-PAGE, and immunoblotted with an antibody against VP1. (B) Large T antigen (TAg)-positive cells were counted in SV40-infected cells treated with or without BFA at infection (0 h.p.i.), and the results reported as the % of TAg expressing cells. Data represent the mean +/− SD of at least 3 independent experiments. In a field of view, 345/378 cells were scored TAg-positive in control cells, while 0/344 cells were scored TAg-positive for BFA-treated cells. (C) A schematic diagram of the ER-to-cytosol transport assay and the ensuing fractionation strategy. (D) Cells treated with or without BFA were incubated with SV40 for the indicated amount of time and processed according to Figure 1C. 10% of P1 and 20% of S1 were loaded. (E) As in D, except BFA was added to cells 4 h.p.i. (F) As in D, except nocodazole was added to cells 0 h.p.i. and the cells harvested 12 h.p.i. (G) Cells transfected with either a scrambled or ERp57 siRNA were infected with SV40 for 12 hrs and processed as in D. (H) SV40-infected cells were harvested at the indicated post-infection time points, lysed in SDS sample buffer and analyzed by immunoblotting with antibodies against TAg, VP1, and Hsp90. Arrow head indicates the initiation time point of TAg synthesis, while arrow indicates the initiation time point of VP1 synthesis. (I) Cells treated with or without BFA at infection were infected with SV40 for 12 hrs and processed as in D. In addition, the S1 was subjected to PCR to amplify a part of the SV40 genome. *(*J) As in I except where indicated, a VP2/VP3 antibody was used.

We first tested whether brefeldin A (BFA), a drug that can impede COPI-dependent retrograde transport from the cell surface to the ER, blocks arrival of SV40 to the ER and infection as reported previously [11], [13]. A convenient method to measure SV40 ER arrival is to monitor conformational changes imparted on the virus in the ER. For instance, when SV40 arrives in the ER, ER-resident protein disulfide isomerase (PDI) family members disrupt its disulfide bonds [12]. When a whole cell extract (WCE) derived from infected cells was analyzed by non-reducing SDS-PAGE, VP1 monomer was detected [12]. Accordingly, simian CV-1 cells were incubated with SV40 (m.o.i. 30) for 12 hrs at 37°C. The cells were solubilized with SDS to generate a WCE, and the samples analyzed by non-reducing SDS-PAGE followed by immunoblotting with VP1-specific antibodies. We detected formation of both VP1 monomer and a species whose size corresponds to a VP1 dimer (Figure 1A, lane 1). An additional VP1 species at the top of the gel was also detected, which is likely derived from the intact virus. The VP1 monomer and dimer levels decreased when cells were treated with BFA at infection (0 h.p.i.) (Figure 1A, compare lane 2 to 1). A similar VP1 monomer level was observed when the samples were subjected to reducing SDS-PAGE (Figure 1A, compare lanes 3 and 4).

BFA was added to cells 4 hrs post infection (4 h.p.i.) to avoid perturbing viral entry. After 8 additional hrs, cells were harvested and analyzed as above. Under this condition, we found that the VP1 monomer and dimer levels also decreased when compared to control cells (Figure S1A, top panel, compare lane 2 to 1), indicating that BFA likely acted at an intracellular step required for ER sorting. Analyses using confocal microscopy further demonstrated that when cells were treated with BFA 4 h.p.i., co-localization between SV40 (green) and ER (red) decreased (Figure S1B, compare right and left panels). Collectively, these results indicate that ER transport is required to generate VP1 monomer and dimer.

To assess BFA's effect on viral infection, control and BFA-treated cells were incubated with SV40, and immunofluorescence microscopy was used to score expression of the virally encoded T antigen (TAg) in the nucleus as before [14]. We found that BFA decreased SV40 infection potently (Figure 1B). This result demonstrates that ER transport is critical for SV40 infection, consistent with previous observations [11], [13]. Thus BFA blocks SV40 trafficking to the ER and infection.

To establish an ER-to-cytosol transport assay for SV40, outlined in Figure 1C, we modified our semi-permeabilized cell-based assay developed previously to probe translocation of cholera toxin (CT) from the ER to the cytosol [15]. In this modified assay, SV40-infected CV-1 cells were treated with a low digitonin concentration (0.1%) to gently permeabilize the plasma membrane while leaving intracellular membranes, including the ER membrane, intact (Figure 1C, step 1). The permeabilized cells were centrifuged at medium-speed (16,000 g) to generate two fractions: a supernatant fraction (S1) that should contain cytosolic proteins, virus that reached the cytosol from the ER, and any endosomal vesicles harboring virus that did not sediment at the medium-speed spin, and a pellet fraction (P1) that should contain the plasma membrane, intracellular organelles including the ER and nucleus, and SV40 that either did not undergo ER-to-cytosol transport or did but is further imported into the nucleus. P1 contents were extracted by Triton X-100 and SDS. When S1 and P1 were subjected to reducing SDS-PAGE followed by immunoblotting, we found the cytosolic marker Hsp90 is predominantly in the S1 (Figure 1D, compare second and fifth panels from top), while the ER lumenal protein PDI was present only in the P1 (Figure 1D, compare 6^th^ and 3^rd^ panels from top). Similar to Hsp90, the cytosolic protein actin also appeared in S1 but not P1 using this fractionation method (Figure S1C, top and bottom panels, compare lane 1 to 2). Hence, this one-step fractionation procedure efficiently separates cytosolic from ER contents, similar to our previous report [15].

When cells were incubated with wild-type (WT) SV40 at 4°C, a condition that blocks endocytosis, and the cells subjected to the fractionation procedure, no VP1 was detected in the S1 (Figure 1D, lane 1, compare first and fourth panels from top). In contrast, when the cells were incubated with SV40 at 37°C for 8 hrs (8 h.p.i.) to allow entry, a portion of VP1 was found in the S1 (Figure 1D, lane 2, compare first and fourth panels from top). When cells treated with BFA at infection (0 h.p.i.) were incubated with SV40 at 37°C for 8 hrs, the VP1 level present in the S1 decreased (Figure 1D, top panel, compare lanes 3 to 2). Similar results were observed when cells were incubated with SV40 at 37°C for 10 hrs and 12 hrs: for both time points, appearance of SV40 in the S1 was blocked significantly by BFA (Figure 1D, top panel, compare lanes 5 to 4 and lanes 7 to 6). Moreover, when BFA was added to cells 4 h.p.i. and the cells harvested after 8 additional hours, the S1 VP1 level also decreased significantly (Figure 1E, top panel, compare lane 2 to 1). Thus, by blocking ER arrival (Figure 1A, S1A, and S1B), BFA also attenuates the subsequent ER-to-cytosol transport of SV40.

We showed previously that BFA also blocked ER-to-cytosol transport of CT [15], [16]. To intoxicate cells, CT via its B subunit (CTB) binds to GM1 on the cell surface, becomes rapidly endocytosed into invaginating vesicles, transported to the early and recycling endosomes, then followed by retrograde sorting through the Golgi and to the ER [17]. In the ER, the catalytic CTA1 undergoes ER-to-cytosol translocation to reach the cytosol where the toxin induces cytotoxicity. We had demonstrated that BFA blocked ER-to-cytosol transport of CTA1 in both HeLa [15] and 293T [16] cells. Here, when CV-1 cells treated with BFA at intoxication were subjected to the semi-permeabilized assay (Figure 1C), the S1 CTA1 level (analyzed 90 min post-intoxication) was significantly decreased when compared to control cells (Figure S1D, top panel, compare lane 1 to 2). This finding is consistent with our previous findings [15], [16] and further substantiates BFA's ability to generally perturb ER-to-cytosol transport processes by disrupting ER arrival.

As SV40 also relies on a nocodazole-sensitive step to reach the ER critical for infection [8], we showed that when cells were treated with nocodazole at infection, the S1 VP1 level 12 h.p.i. was blocked completely when compared to control cells (Figure 1F, top panel, compare lane 1 to 2). Hence nocodazole effectively perturbed SV40's ER-to-cytosol transport, presumably by blocking viral transport to the ER.

A more detailed time-course experiment using the semi-permeabilized system demonstrated that significant VP1 level started to appear in the S1 approximately 6 h.p.i., although a low VP1 level appeared in the S1 at 4 h.p.i. (Figure S1E, top panel). Because a previous study demonstrated that SV40 arrives to the ER approximately 6 h.p.i. [18], the low VP1 level in the S1 at 4 h.p.i. is unlikely virus that underwent ER-to-cytosol transport. Instead, it may represent virus that either leaked from a membrane compartment due to digitonin treatment or in transport vesicles en route to the ER which did not pellet after medium-speed centrifugation.

To test the former possibility, we asked whether digitonin causes leakage of CTB from membrane vesicles. CTB is used because it is much smaller than SV40, binds to ganglioside GM1 (akin to VP1), and is also targeted to the ER similar to SV40. Accordingly, cells were intoxicated with CT for either 5 min (where CTB is found in vesicles/endosomes) or 90 min (where CTB is found in a mixture of endosomes, Golgi, and ER). Following digitonin treatment, cells were subjected to 16,000 g medium-speed centrifugation to generate S1 (Figure S1F, see diagram and top and bottom panels, lane 1). S1 was treated with or without 2% SDS and subjected to high-speed centrifugation (100,000 g) to generate a supernatant (sn) and pellet fractions. Under this condition, vesicles harboring CTB should pellet, while CTB that leaked due to membrane rupture by digitonin should appear in the sn. We found that, at both time points, CTB appeared only in the pellet but not the sn (Figure S1F, top and bottom panels, compare lane 5 to 3). If SDS was added to S1 to artificially solubilize vesicles prior to high-speed centrifugation, CTB appeared in the sn but not pellet instead (Figure S1F, top and bottom panels, compare lane 6 to 4). We conclude that digitonin treatment did not cause CTB leakage from vesicles. Thus, because CTB is much smaller than SV40, it is unlikely that digitonin disrupted any membrane vesicles to cause leakage of SV40.

To test the idea that VP1 in the S1 at 4 h.p.i. represents SV40 in transport vesicles that did not sediment after medium-speed centrifugation, we first used limited proteolysis because this method distinguishes between membrane-encased virus versus naked virus. Because of the low VP1 level in the S1 at 4 h.p.i., a higher amount of this sample was used to visualize VP1. We found that VP1 in the S1 at 4 h.p.i. is resistant to trypsin digestion, in contrast to virus at 12 h.p.i. (Figure S1G, compare top and bottom panels, lanes 1 to 2 and 3). These findings indicate that SV40 in the S1 at 4 h.p.i. is likely contained in membrane vesicles, while those at 12 h.p.i. are not.

To further support this view, we subjected SV40 in the S1 at both 4 and 12 h.p.i., as well as purified WT SV40, to OptiPrep gradient flotation. The majority of VP1 at 4 h.p.i. floated to lighter density fractions when compared to purified SV40 (Figure S1H, compare top and bottom panels). In contrast, VP1 at 12 h.p.i. displayed very little flotation when compared to purified SV40 (Figure S1H, compare middle and bottom panels). These results demonstrate that the low SV40 level in the S1 at 4 h.p.i. is membrane-bound, presumably reflecting transport vesicles carrying SV40 that have not arrived to the ER. By contrast, virus at 12 h.p.i. is naked and not in vesicles, consistent with the property of a viral particle that has penetrated the ER membrane. We conclude that VP1 in the S1 at the 12 h.p.i. time point, as well as at the earlier 8 and 10 h.p.i. time points (see below), represents the virus pool that reached the cytosol from the ER.

An increase in cytosol-localized SV40 should allow more viral particles to enter the nucleus to cause infection. We found that increasing the m.o.i. increased both the S1 VP1 level at 12 h.p.i. (Figure S1I, top panel, lanes 1–6) and infection (Figure S1I, bottom graph). This correlation is consistent with the view that virus in S1 at 12 h.p.i. represents cytosol-localized virus poised to enter the nucleus to promote infection.

To further verify that the semi-permeabilized assay reflects SV40's ER-to-cytosol transport, we reasoned that down-regulation of ER-resident factors implicated in SV40 infection should block ER-to-cytosol transport as well. As ERp57 down-regulation decreased virus infection [12], we showed that ERp57 knock-down also decreased the S1 VP1 level at 12 h.p.i. (Figure 1G, top panel, compare lane 1 to 2). Similarly, we found that down-regulation of a novel ER-resident DNA J protein required for efficient SV40 infection also decreased the amount of S1 VP1 (manuscript in preparation). Finally, as treating cells with dithiothreitol (DTT) was shown to attenuate infection [12], we found that DTT treatment decreased both the S1 SV40 level (at 12 h.p.i.) and infection (Figure S1J, top panel, compare lane 2 to 1, and right graph). These findings further validate the semi-permeabilized system as an ER-to-cytosol transport assay.

In CV-1 cells, the earliest expression of new VP1 occurred at 20 h.p.i. (Figure 1H, middle panel, arrow), consistent with an earlier report in the same cell line [19]. This finding demonstrates that VP1 in the S1 derived from cells incubated with SV40 for 8, 10, and 12 hrs (Figure 1D, top panel, lanes 2, 4, and 6) is input but not de novo synthesized virus. We note that TAg expressed at 14 h.p.i. (Figure 1H, top panel, arrow head), suggesting that only a small proportion of virus in the P1 at the 8, 10, and 12 h.p.i. time points represents nuclear-localized virus. When control and BFA-treated cells were incubated with a biotinylated SV40 for 12 hrs, and the cells subjected to the ER-to-cytosol transport assay, biotinylated VP1 (as detected by streptavidin binding) was detected in the S1 derived from control and to a lesser extent BFA-treated cells (Figure S1K, top panel, compare lane 1 to 2). This finding further proves that the input virus reaches the cytosol.

Do other viral components undergo ER-to-cytosol transport? In addition to immunoblotting, the S1 from control and BFA-treated cells infected with SV40 for 12 hrs were subjected to PCR analyses using primers designed to amplify an SV40 genome fragment. We found presence of the viral genome in S1 derived from control but not BFA-treated cells (Figure 1I, top panel, compare lane 1 to 2). Similarly, using a VP2/VP3-specific antibody, we detected VP2 and VP3 in S1 derived from control but not BFA-treated cells (Figure 1J, top panel, compare lane 1 to 2). The higher VP3 intensity when compare to VP2 is not due to preferential antibody binding to VP3 as VP2 contains all of VP3 except VP2 has an additional N-terminal extension. Instead, this observation is likely because the input SV40 particle contains more VP3 than VP2 (below), similar to a previous report [20]. These results demonstrate that VP2, VP3, and the viral genome are co-transported with VP1 from the ER to the cytosol.

### ER-localized SV40 is released into the ER lumen, and undergoes sequential disulfide bond disruption to reach the cytosol

We next analyzed ER events that prime SV40 for membrane penetration by taking further advantage of the semi-permeablized system. We hypothesize that, upon ER arrival, SV40 remains bound to GM1 on the lumenal surface of the ER membrane, as the related mPy associates with its ganglioside receptor GD1a when this virus reaches the ER [10]. We postulate that SV40 is next released into the lumen by detaching from GM1. Here it undergoes conformational changes that enable the virus to re-engage the ER membrane, ultimately penetrating this bilayer to reach the cytosol. At steady state, there should be a virus pool attached to GM1 on the ER membrane, in the ER lumen, trapped on the ER membrane in the act of penetration, and in the cytosol. Analyzing specific SV40 conformations in each pool should reveal the sequence of events and the mechanism guiding membrane penetration.

P1 in our assay ought to contain SV40 attached to GM1 on the ER membrane (as well as on the plasma membrane and other organelles), in the ER lumen, and trapped on the ER membrane in transit to the cytosol. In contrast, S1 should contain virus that reached the cytosol (or in transport vesicles at the earlier time point). Because GM1 is enriched in membrane microdomains referred to as lipid rafts [18], SV40 attached to GM1 should localize to lipid rafts. Contents in this microdomain are often found to be resistant to Triton X-100 extraction [21]. Thus, SV40 that reaches the ER but remains bound to GM1 is resistant to Triton X-100 extraction, while those virus released into the ER lumen or trapped on the ER membrane en route to the cytosol are extracted by this detergent. Contents resistant to Triton X-100 extraction can be extracted by SDS.

**Figure 2:**
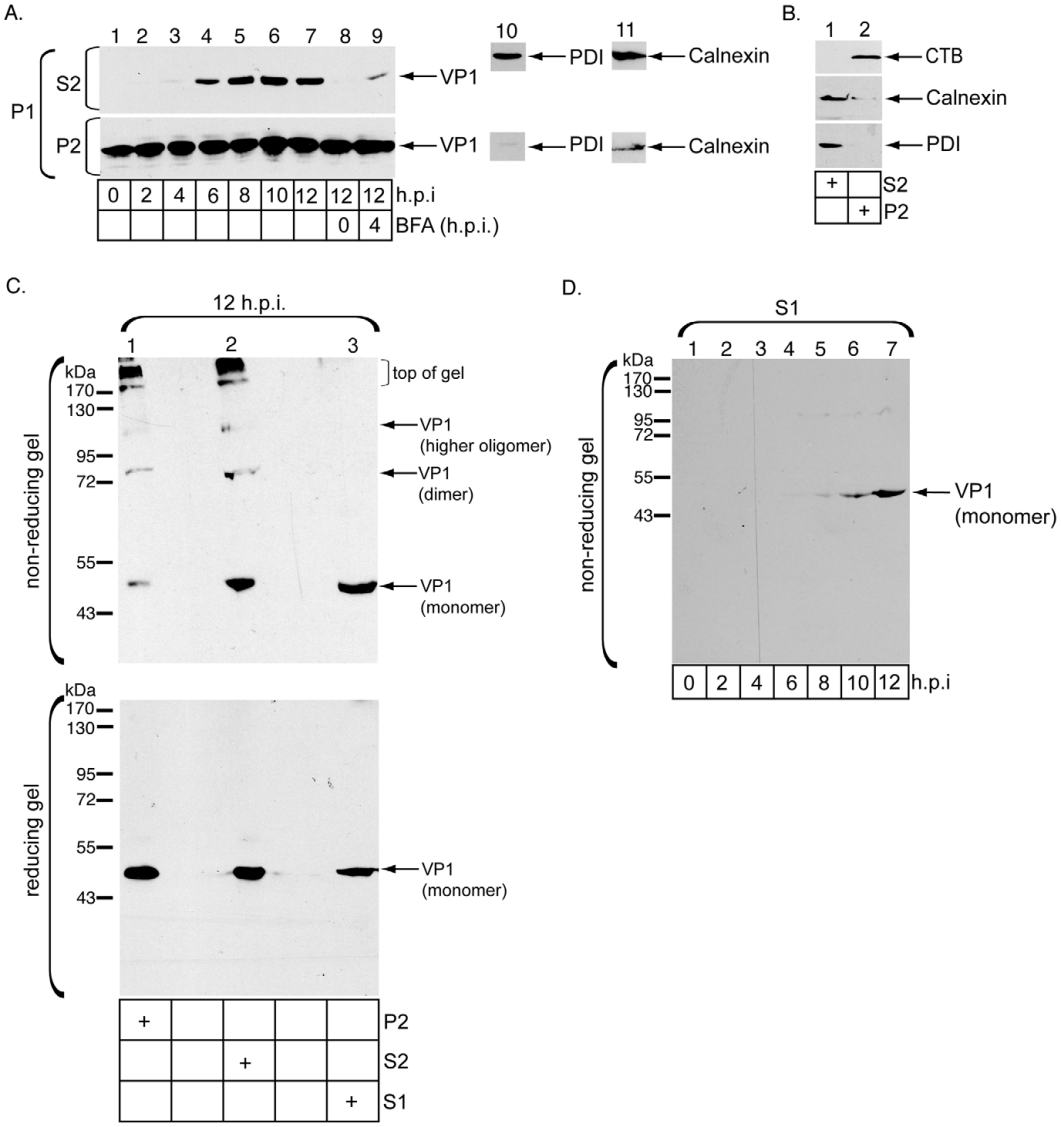
ER-localized SV40 is released into the ER lumen and undergoes sequential disulfide bond disruption. (A) Cells treated with or without BFA at the indicated time points were infected with SV40 for varying amounts of time, harvested, and analyzed according to Figure 1C. Samples were immunoblotted with antibodies against VP1, PDI, or calnexin. (B) Cells were intoxicated with CTB for 90 min, and processed according to Figure 1C. Samples were immunoblotted with antibodies against PDI, calnexin, and CTB. (C) Cells were infected with SV40 and harvested at 12 h.p.i. S1, S2, and P2 were prepared as in A and analyzed by non-reducing and reducing SDS-PAGE, followed by immnoblotting with antibodies against VP1. (D) Cells were infected with SV40 for the indicated times, and S1 subjected to non-reducing SDS-PAGE followed by immunoblotting against VP1.

Accordingly, P1 derived from cells incubated with SV40 for varying times were solubilized with Triton X-100 (Figure 1C, step 2). After centrifugation, the resulting supernatant contains the Triton X-100 extractable material (S2), while the new pellet contains Triton X-100 insoluble material that was extracted by SDS (P2). The S2 and P2 samples were subjected to immunoblot analysis. We found that while VP1 is present in P2 throughout the entire course of the experiment (Figure 2A, bottom panel, lanes 1–7), VP1 only appeared in the S2 starting at 6 h.p.i. (Figure 2A, top panel, compare lanes 4–7 to lanes 1–3). Under these conditions, PDI and most of the ER membrane protein calnexin are found in S2 but not P2 (Figure 2A, lane 9 and 10, compare top and bottom panels), as expected for an ER lumenal and membrane protein not enriched in lipid rafts.

VP1's appearance in S2 derived from cells incubated with virus for 12 hrs is blocked completely when cells are pretreated with BFA (Figure 2A, top panel, compare lane 8 to 7). S2 VP1 also decreased significantly if BFA is added 4 h.p.i. (Figure 2A, top panel, compare lane 9 to 7), again demonstrating that BFA blocked an intracellular step important for SV40 sorting to the ER. As a control, we found that CTB, which is also found in lipid raft-enriched membranes, remains exclusively in the P2 and not S2 (Figure 2B, top panel, compare lane 2 to 1), indicating that Triton X-100 did not non-specifically disrupt lipid raft membrane domains to release SV40. These results demonstrate that ER transport is required to generate Triton X-100-extractable virus, consistent with the hypothesis that SV40 detaches from GM1 upon ER arrival. Thus, while SV40 in P2 represents virus concentrated in membrane rafts due to its interaction with GM1, SV40 in S2 represents virus that reached the ER and is released into the ER lumen, either preparing for membrane penetration or trapped on the ER membrane in transit to the cytosol. SV40's appearance in the ER starting at 6 h.p.i. in this assay is in agreement with previous studies [12], [18], and is consistent with the notion that SV40 arrives in the cytosol after 6 h.p.i. (Figure S1E, top panel).

P2, S2, and S1 contain SV40 at different stages of membrane penetration. To examine the nature of SV40's disulfide bonds in these fractions, samples from the three fractions generated from cells infected with SV40 for 12 hrs were subjected to non-reducing SDS-PAGE followed by immunoblotting with VP1-specific antibodies. VP1 monomer, dimer, and virus at top of the gel were detected in P2 (Figure 2C, top panel, lane 1). In S2, a faint species corresponding to a VP1 higher oligomer, dimer, and more monomer (when compared to its P2 level) were observed (Figure 2C, top panel, lane 2). By contrast, only VP1 monomer was detected in S1 (Figure 2C, top panel, lane 3). When all three fractions were subjected to reducing SDS-PAGE, VP1 monomer was the only species observed (Figure 2C, bottom panel, lanes 1-3).

Thus, when the virus initially arrives in the ER attached to the membrane, disulfide bond disruption is initiated, generating VP1 monomer and dimer (Figure 2C, lane 1). When the virus is released into the ER lumen or becomes subsequently trapped on the ER membrane en route to the cytosol, intact virus is converted to the VP1 higher oligomer, and the dimer is further reduced to the monomer (Figure 2B, compare lane 2 to 1). Finally, upon cytosol arrival, complete disruption of the disulfide bonds ensues, generating VP1 monomer (Figure 2B, compare lane 3 to 2). These results demonstrate a sequential rearrangement of SV40's disulfide bonds as it moves from the ER to the cytosol. We note that as monomer and dimer were not detected in any of the fractions using non-SDS biochemical methods (below), they likely still consist of VP1 pentamers that remain in contact with the core viral particle via non-covalent interactions.

As complete disruption of disulfide bonds that generates VP1 monomer (in a non-reducing SDS condition) is a hallmark of cytosol-localized SV40, we performed a time-course experiment using a non-reducing SDS-PAGE and showed that VP1 monomer appeared in S1 at approximately 8 h.p.i. (Figure 2D, lanes 5–7). These findings further support the assertion that SV40 begins to arrive to the cytosol sometime after 6 h.p.i., also consistent with our measurement of SV40 ER arrival at approximately 6 h.p.i.

### Immunoprecipitation of ER- and cytosol-localized SV40 using conformation-specific antibodies

The disulfide bond arrangement of ER- and cytosol-localized SV40 is distinct (Figure 2B, top panel, compare lane 2 to 3). However, whether this difference affects the global viral conformation is unknown. We therefore evaluated the virus structures in S1 and S2 using four independent biochemical approaches.

We first used conformation-specific antibodies for this purpose. Two monoclonal VP1 antibodies (CC10 and BC11) were shown to neutralize SV40 infection, but did not recognize denatured virus during immunoblotting [22]. We found that these antibodies precipitated the VP1 pentamer (not shown). Hence, the CC10 and BC11 antibodies recognize structural features of the intact pentamer, but not unfolded virus whose epitopes critical for antibody recognition are disordered. We reasoned that, at a sub-saturating antibody concentration where there is insufficient antibody to bind to all available VP1, a given CC10 or BC11 antibody should precipitate more VP1 if the virus is assembled and intact than disassembled and uncoated. In contrast, at a saturating antibody concentration, a similar VP1 level would be precipitated by the antibodies regardless of the viral structural state. Thus, using antibodies at a sub-saturation condition could potentially reveal the global structural state of SV40.

**Figure 3:**
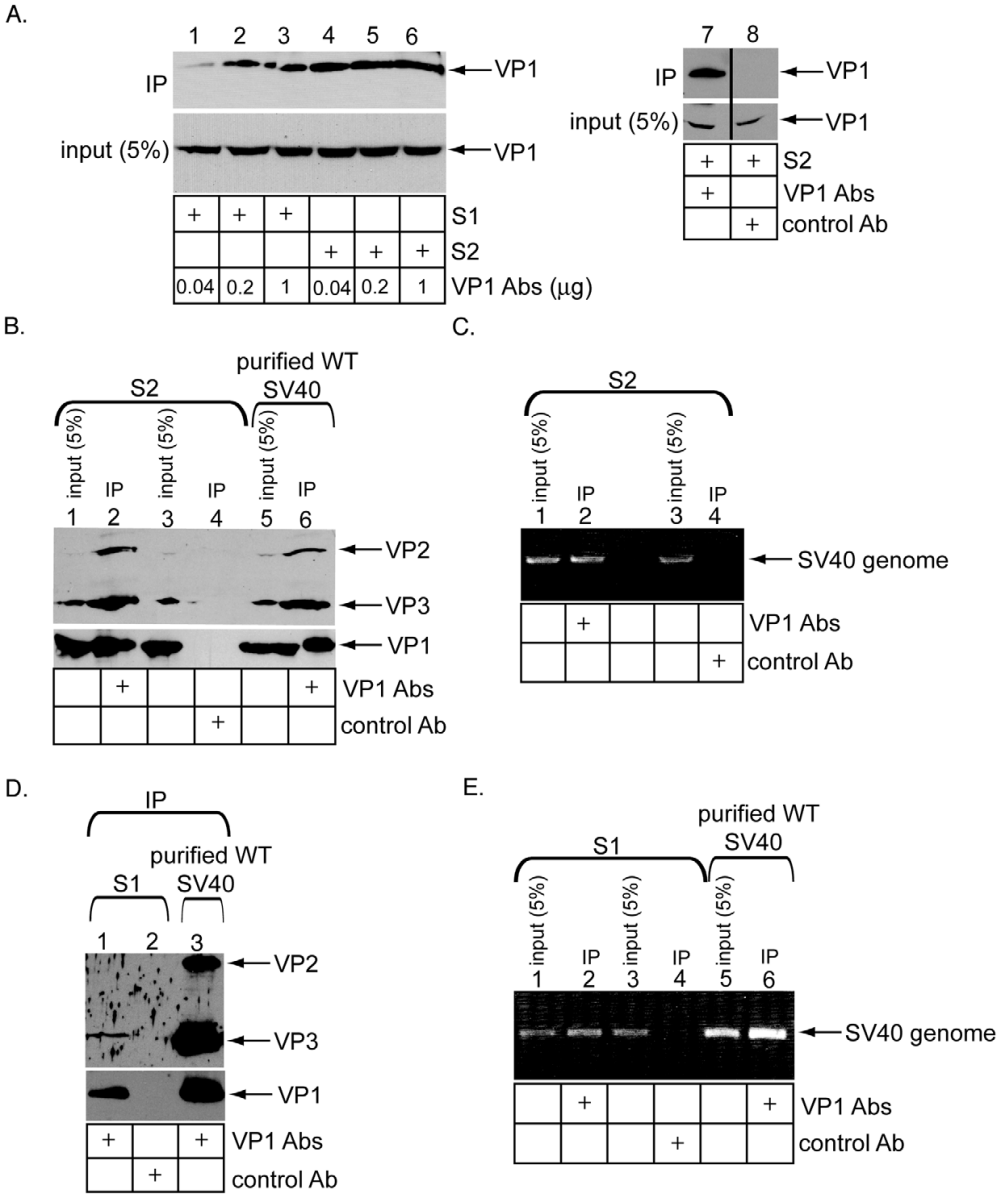
Immunoprecipitation of ER- and cytosol-localized SV40 using conformation-specific antibodies. (A) Cells were infected with SV40 for 12 hrs, harvested, and processed according to Figure 1C to obtain S1 and S2. These fractions were incubated with the indicated VP1 antibody concentration, or a control antibody. The precipitated samples were subjected to SDS-PAGE followed by immunoblotting against VP1. 5% input is shown. (B) S2 in A and WT SV40 were subjected to immunoprecipitation using either VP1 or a control antibody, and the precipitated sample subjected to SDS-PAGE and immunoblotted with antibodies against VP2/VP3 or VP1. 5% input is shown. (C) The inputs and immunoprecipitates in B were subjected to PCR to amplify a region of SV40 genome. 5% input is shown. (D) As in B, except the S1 was used for immunoprecipitation. (E) As in C, except the S1 was used as the starting material.

Accordingly, at 12 h.p.i., cells were subjected to the semi-permeabilized assay, and virus in S1 and S2 immunoprecipitated with a mixture of increasing amounts of the VP1 monoclonal antibodies. VP1 in S1 precipitated less efficiently than VP1 in S2 when a low (i.e. 0.04 µg) level of antibodies was used (Figure 3A, top panel, compare lane 1 to 4). However, the difference in the precipitation efficiency gradually disappeared when higher levels of antibodies (i.e. 0.2 and 1 µg) were used (Figure 3A, top panel, compare lanes 2 and 3 to lanes 5 and 6). A control antibody did not precipitate VP1 from S2 (Figure 3A, top panel, lane 8). Thus, in our experimental conditions, 0.04 µg represents a sub-saturating antibody concentration in which differences between the structural organization of SV40 in S1 and S2 can be revealed. Specifically, that 0.04 µg of the SV40 antibodies precipitated less VP1 from S1 than S2 suggests that virus in S1 underwent disassembly.

VP2/VP3 in S2 co-precipitated with VP1 specifically (Figure 3B, top panel, compare lane 2 to 4), with an efficiency similar to that observed when purified WT SV40 was used as the starting material (Figure 3B, top panel, compare lane 2 to 6). In addition, the SV40 genome also co-precipitated with VP1 from S2 specifically (Figure 3C, compare lane 2 to 4). In contrast, VP2 and VP3 in S1 co-precipitated weakly with VP1 when compared to the efficiency observed using purified WT SV40 (Figure 3D, top panel, compare lane 1 to 3), even when 5-fold more S1 than S2 was used for immunoprecipitation. The SV40 genome co-precipitated with VP1 in S1 specifically (Figure 3E, compare lane 2 to 4). Our results suggest that the ER-localized SV40 is more assembled and intact than the cytosol-localized virus, and retains strong binding to the internal viral components. The cytosol-localized virus likely experienced disassembly, and displays less interaction with its internal proteins.

### ER-localized SV40 is large, while those in the cytosol are large and small

**Figure 4:**
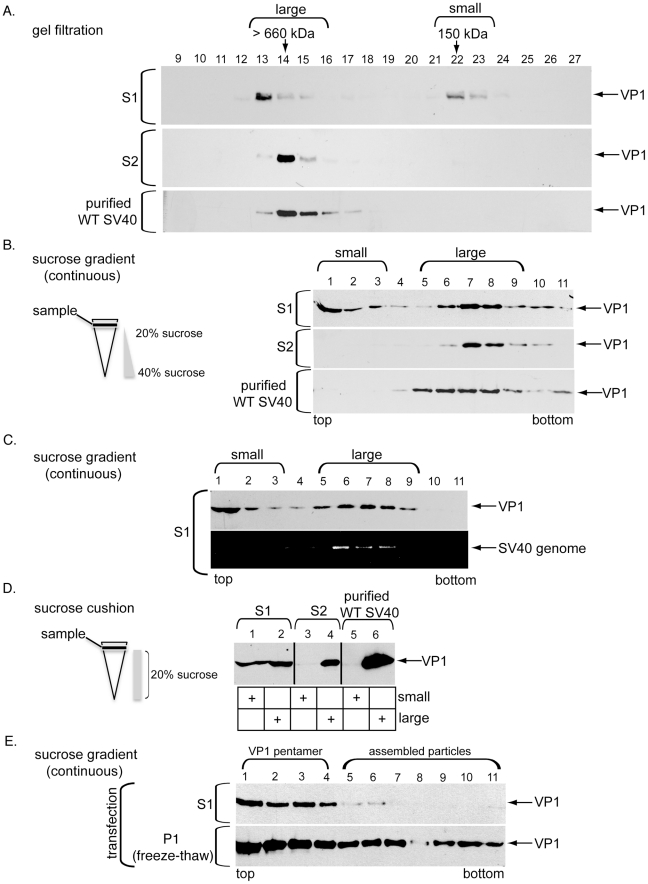
ER-localized SV40 is large, while cytosol-localized SV40 is large and small. (A) S1 and S2 derived from cells infected with SV40 for 12 hrs, and purified SV40, were subjected to gel filtration. Fractions were analyzed by SDS-PAGE, followed by immunoblotting with antibodies against VP1. VP1 was detected in two peak fractions, “large” (fractions 13–15) and “small” (fractions 22–23). (B) S1, S2, and purified SV40 in A were subjected to continuous sucrose gradient centrifugation and analyzed as in A. VP1 was mainly detected in two peak fractions, “small” (fractions 1–3) and “large” (fractions 5–9). (C) S1 in A was subjected to sucrose gradient centrifugation. Fractions were analyzed as in A and subjected to PCR to amplify a part of SV40 genome. (D) As in B, except samples were layered over a 20% sucrose cushion, centrifuged, and the sedimented material (large) and material near the top of the cushion (small) was analyzed by immunoblotting. (E) Cells were transfected with an SV40 genome and processed as in B, except the pellet was subjected to repeated freeze-thaw to extract the nuclear-localized virus.

As a second method to probe SV40's conformations in the ER and cytosol, S1 and S2 prepared from cells infected with SV40 for 12 hrs were subjected to gel filtration analyses. Our data showed that essentially all the viral particles in S2 are found in fractions similar to purified WT SV40 (estimated to be >660 kDa in our system due to resolution of the column) (Figure 4A, compare second and third panels from top). For simplicity, these viral particles are referred to as “large” particles (Figure 4A). In contrast, a virus pool in S1 was found in fractions that corresponded to “small” particles approximating 150 kDa, while another portion was located in fractions corresponding to the large particle (Figure 4A, top panel). The 150 kDa species likely represents the VP1 pentamer. These results demonstrate that all the SV40 particles in the ER are large, while virus in the cytosol exists as large and small particles.

We next used continuous (20–40%) sucrose gradient sedimentation as a third approach to examine SV40's structure in the ER and cytosol (Figure 4B). Again, whereas all the virus in S2 sedimented to bottom heavier fractions similar to purified WT SV40 corresponding to the large particle (Figure 4B, compare second and third panels from top), a portion of virus in S1 was found in the top lighter fractions corresponding to the small particle and another portion in the heavier fractions corresponding to the large particle (Figure 4B, top panel). The virus remained in these lighter fractions even when S1 was pretreated with Triton X-100 prior to sedimentation (not shown), indicating that SV40 in these fractions is not due to flotation caused by membrane encapsulation. PCR analysis further demonstrated that the large but not small viral particles in S1 contain the viral genome (Figure 4C, compare bottom and top panels). This result is consistent with our co-immunoprecipitation analysis demonstrating that the cytosol-localized SV40 binds to the genome (Figure 3E).

To estimate the proportion of SV40 in S1 and S2 that are small and large, these samples (along with purified WT SV40) were layered over a sucrose cushion (20%) and centrifuged (Figure 4D). The large particle is expected to penetrate the sucrose cushion and sediment, while the small particles should remain near the top of the cushion. When the sedimented material (labeled large) and material near the top of the cushion (labeled small) were subjected to immunoblotting, approximately 50% of virus in S1 were found in the small fraction and 50% in the large fraction (Figure 4D, compare lane 1 to 2). In contrast, essentially all of the virus in S2 and a sample containing purified WT SV40 was large (Figure 4D, compare lane 4 to 3 and 6 to 5). This size distribution is consistent with the gel filtration (Figure 4A) and continuous sucrose sedimentation (Figures 4B and 4C) findings.

Results using four distinct biochemical strategies (i.e. immunoprecipitation, gel filtration, continuous sucrose gradient sedimentation, and sucrose cushion sedimentation) demonstrate unambiguously that SV40 in the ER is a large particle, while the virus in the cytosol exists as small and large particles. The simplest explanation of these findings is that ER-localized SV40 penetrates the ER membrane as a large and intact particle, reaching the cytosol where it disassembles into small particles. The remaining core particle after cytosol-mediated disassembly, which remains relatively large and cannot be distinguished from the large ER-localized particle using either gel filtration or sucrose gradient analysis, contains the genome and is likely the predecessor to the form that enters the nucleus.

Alternatively, it is possible that the ER-localized large particle disassembles into small particles in the ER, become discharged into the cytosol where they re-assemble into a large particle. To test whether the cytosol supports large particle assembly in our system, we analyzed SV40 virion formation by transfecting cells with the viral genome. Using this method, VP1 monomer should be made in the cytosol, followed by its oligomerization into pentamers in this compartment. The pentamers are expected to import into the nucleus for full assembly into the large SV40 particle. We found that when cells were transfected with the SV40 genome for 48 hrs, subjected to the semi-permeabilized assay, and the S1 and P1 analyzed by sucrose gradient sedimentation, only small particles were found in the S1 (Figure 4E, top panel, fractions 1–4). These small particles represent the cytosol-localized pentamers. By contrast, VP1 appeared in virtually all fractions in the P1 (Figure 4E, bottom panel). (The pellet was subjected to repeated freeze-thaw to extract virus from the nucleus). VP1 in the top fractions corresponds to nuclear-localized pentamers imported from the cytosol while those in the heavier fractions correspond to viral particles in the nucleus undergoing assembly. Thus, when cells were transfected with the SV40 genome, VP1 pentamers are generated in the cytosol and imported into the nucleus to form large particles, consistent with the established SV40 assembly process [20]. Importantly, these results demonstrate that the cytosol does not support large particle formation from small particles.

### Negative stain electron microscopy (EM) of cytosol-localized SV40

**Figure 5:**
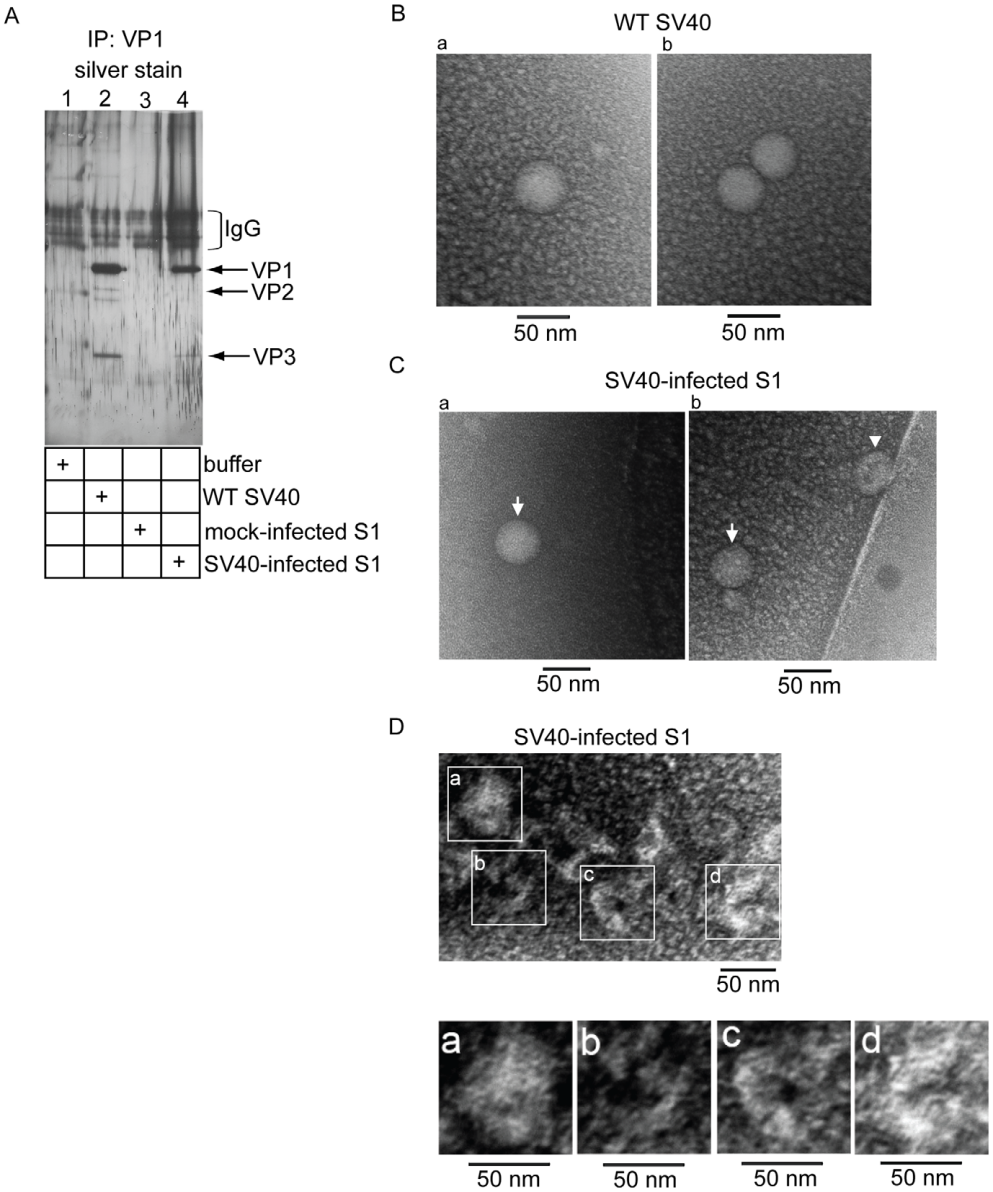
Negative stain EM of WT and cytosol-localized SV40. (A) Buffer, WT SV40, S1 derived from mock-infected cells (i.e. mock-infected S1), and S1 derived from SV40-infected cells for 12 hrs (i.e. SV40-infected S1) were immunoprecipitated with VP1-specific antibodies, and the immunoprecipitate captured by magnetic beads. Samples were subjected to SDS-PAGE and silver stained. (B) WT SV40 immunoprecipitate was subjected to negative stain EM. Bar represents 50 nm. (C-D) SV40-infected S1 immunoprecipitate was subjected to negative stain EM. Bar represents 50 nm.

We next sought to visualize the large SV40 particle in the S1 cytosol. Buffer, WT SV40, S1 derived from mock-infected cells (i.e. mock-infected S1), and S1 derived from SV40-infected cells for 12 hrs (i.e. SV40-infected S1) were immunoprecipitated with VP1-specific antibodies, and the immunoprecipitate captured by magnetic beads. Samples were subjected to SDS-PAGE and silver stained. A distinct band corresponding to VP1 was detected in samples derived only from WT SV40 and SV40-infected S1 (Figure 5A, lanes 2 and 4). In addition, a band corresponding to VP3 was also found in the WT SV40 and SV40-infected S1 immunoprecipitate (Figure 5A, lanes 2 and 4), consistent with the co-immunoprecipitation result presented in Figure 3D. When the immunoprecipitate derived from WT SV40 was subjected to negative stain EM, a mostly homogenous population of spherical particles approximately 50 nm could be seen (Figure 5B, a and b).

Interestingly, while spherical particles approximating 50 nm could also be observed in the SV40-infected S1 immunoprecipitate (Figure 5C, a and b (white arrow)), others appeared to be slightly distorted, appearing as elongated spheres with what seems to be pores in the middle (Figure 5C, b (white arrow head)). Even more distorted SV40 particles around 50 nm could also be found in the S1 immunoprecipitate. In these cases, some of their overall structures were poorly defined (Figure 5D, a and d), while others appeared again to be elongated spheres with a doughnut-shaped pore in the middle (Figure 5D, c) or contained a clover leaf-shaped hole (Figure 5D, b). Thus S1 SV40 particles are heterogeneous in structure, and likely represent the large particle pool identified in our biochemical assays.

### Role of VP3 and viral genome in SV40 ER-to-cytosol membrane transport

**Figure 6:**
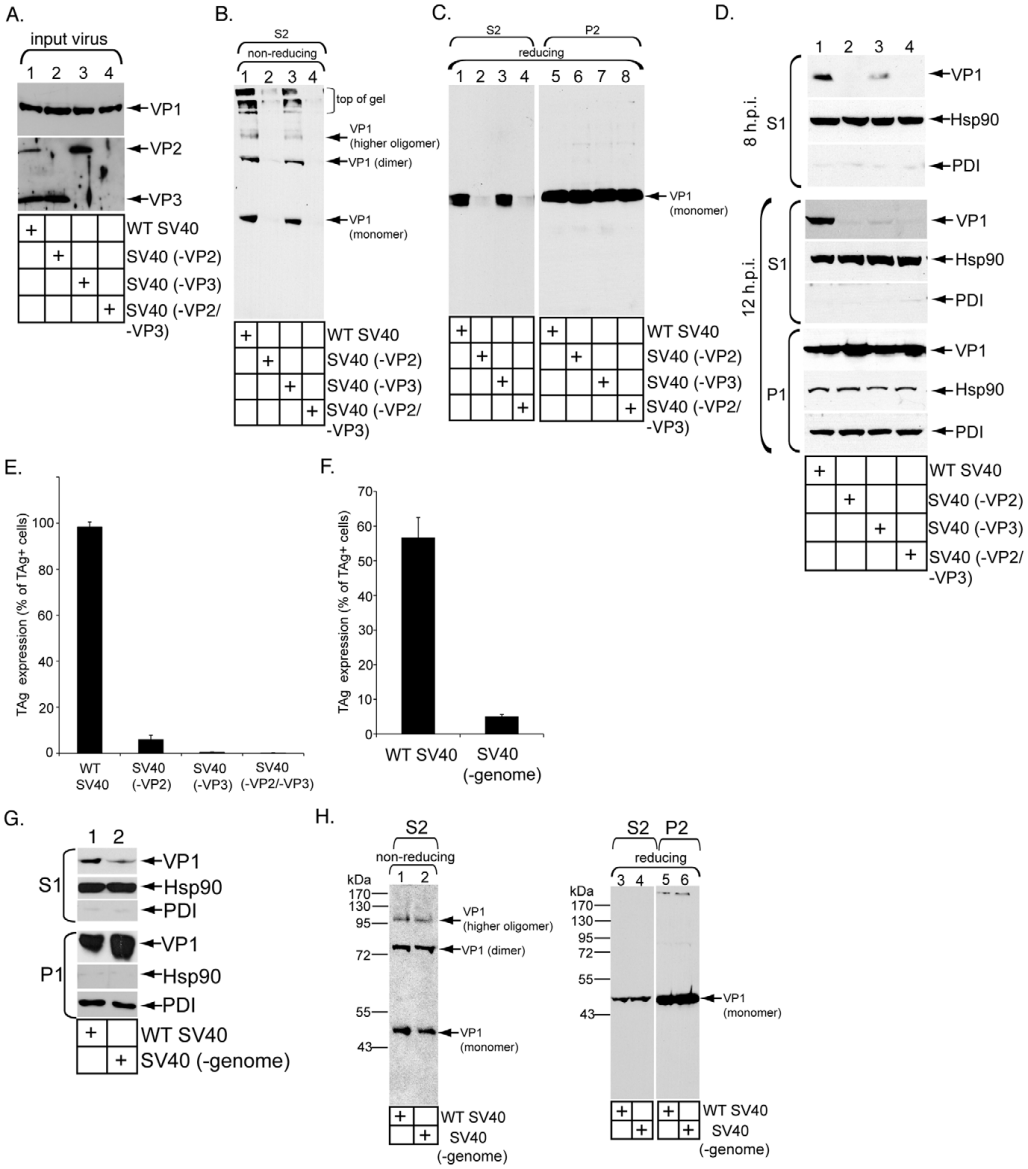
Role of VP3 and viral genome in SV40 ER-to-cytosol membrane transport. (A) WT and mutant viruses lacking VP2, VP3, or both were analyzed by immunoblotting with antibodies against VP1 and VP2/VP3. (B) Cells were infected with the indicated virus for 6 hrs, harvested, and subjected to the semi-permeabilized assay to obtain S2. S2 was subjected to non-reducing SDS-PAGE followed by immunoblotting against VP1. (C) As in B, except both S2 and P2 were prepared. Both fractions were subjected to reducing SDS-PAGE and immunoblotted with a VP1 antibody. (D) Cells were infected with the indicated virus for either 8 or 12 hrs, harvested, and subjected to the ER-to-cytosol membrane penetration assay. S1 (60% of total) and P1 (5% of total) were analyzed by SDS-PAGE, followed by immunoblotting with the indicated antibodies. (E) Cells were infected with the indicated virus and analyzed as in Figure 1B. In a field of view, 467/471 cells scored TAg-positive using WT SV40, 26/387 cells using SV40 (-VP2), 0/375 using SV40 (-VP3), and 0/421 cells using SV40 (-VP2/-VP3). (F) The extent of TAg expression induced by WT and SV40 (-genome) was analyzed as in E. In a field of view, 205/359 cells scored TAg-positive using WT SV40, and 17/379 cells using SV40 (-genome). m.o.i. = 5 was used. (G) Cells were infected with either WT SV40 or SV40 (-genome) for 12 hrs and analyzed as in D. (H) Cells were infected with either WT SV40 or SV40 (-genome) for 12 hrs, and processed to obtain S2 and P2. S2 was subjected to both non-reducing and reducing SDS-PAGE, and P2 was subjected to reducing SDS-PAGE.

In addition to elucidating the ER membrane penetration mechanism, we characterized the viral components regulating this process. As VP2, VP3, and viral genome co-transport with VP1 to the cytosol (Figure 1), we asked whether these internal components control ER-to-cytosol transport. To address whether the minor coat proteins play a role, we generated SV40 mutant viruses lacking VP2 (SV40 (-VP2)), VP3 (SV40 (-VP3)), or both (SV40 (-VP2/-VP3)) (Figure 6A, top and bottom panel, compare lanes 2–4 to 1). VP3's band intensity is higher than VP2 in WT SV40 (Figure 6A, bottom panel, lane 1), indicating more VP3 than VP2 per viral particle, as reported previously [20].

We first determined whether the mutant viruses reach the ER with equal efficiency as WT SV40 by assessing their ability to undergo both ER-dependent disulfide disruption and release from GM1-enriched lipid raft membranes. Cells were incubated with WT or mutant SV40 for 6 hrs, and the S2 prepared. When S2 was subjected to non-reducing SDS-PAGE, SV40 (-VP3) displayed a similar VP1 banding pattern as WT SV40 (Figure 6B, compare lane 3 to 1). In contrast, very low signal was detected in S2 derived from cells infected with SV40 (-VP2) or SV40 (-VP2/-VP3) (Figure 6B, compare lanes 4 and 2 to 1). As expected, when the S2 was subjected to reducing SDS-PAGE, a similar VP1 level was seen between WT and SV40 (-VP3), and essentially no signal was detected from samples derived from SV40 (-VP2) or SV40 (-VP2/-VP3) (Figure 6C, compare lanes 1 and 3 to 2 and 4). The VP1 level was similar in all samples in the P2 (Figure 6C, lanes 5–8), indicating that the total cell-associated virus is the same between WT and mutant viruses. These results demonstrate that SV40 (-VP3), but not SV40 (-VP2) or SV40 (-VP2/-VP3), reaches the ER with similar efficiency as WT SV40 at 6 h.p.i.; SV40 (-VP2) and SV40 (-VP2/-VP3) likely entered the cells but failed to sort to the ER.

We next asked whether the mutant viruses undergo ER-to-cytosol transport by assessing the S1 VP1 level at both 8 and 12 h.p.i. using the semi-permeabilized system described in Figure 1. We found that the S1 VP1 level for all mutant viruses decreased significantly at both time points when compared to WT SV40 (Figure 6D, top and fourth panels, compare lanes 2–4 to 1). The mutant viruses also promoted infection poorly when compared to WT SV40 (Figure 6E). As SV40 (-VP3) reaches the ER from the cell surface with similar efficiency as WT SV40 at 6 h.p.i., we conclude that VP3 plays a critical role in ER-to-cytosol transport. Because SV40 (-VP2) and SV40 (-VP2/-VP3) did not reach the ER, they are expected to not undergo subsequent ER-to-cytosol transport. Thus, our results cannot distinguish a role of VP2 in the ER-to-cytosol penetration process. Of interest, VP2 and VP3 were shown previously to be necessary for nuclear entry [23].

To address the viral genome's role in facilitating ER exit of SV40, we enriched for SV40 that lacked the genome (SV40 (-genome)) on a CsCl gradient. As expected, infection caused by SV40 (-genome) was attenuated severely when compared to WT SV40 (Figure 6F, approximately 9% of WT). When cells incubated with this mutant virus for 12 hrs were subjected to the semi-permeabilized assay, the S1 VP1 level decreased when compared to the VP1 level derived from cells infected with WT SV40 (Figure 6G, top panel, compare lane 2 to 1). SV40 (-genome) and WT SV40 underwent similar ER-dependent disulfide rearrangement (Figure 6H, compare lane 2 to 1) and release from lipid raft membrane domains (Figure 6H, compare lane 4 to 3). We conclude that in addition to VP3, the SV40 genome appears to also mediate its ER-to-cytosol transport.

### SV40 release into the cytosol depends on the host proteasome

**Figure 7:**
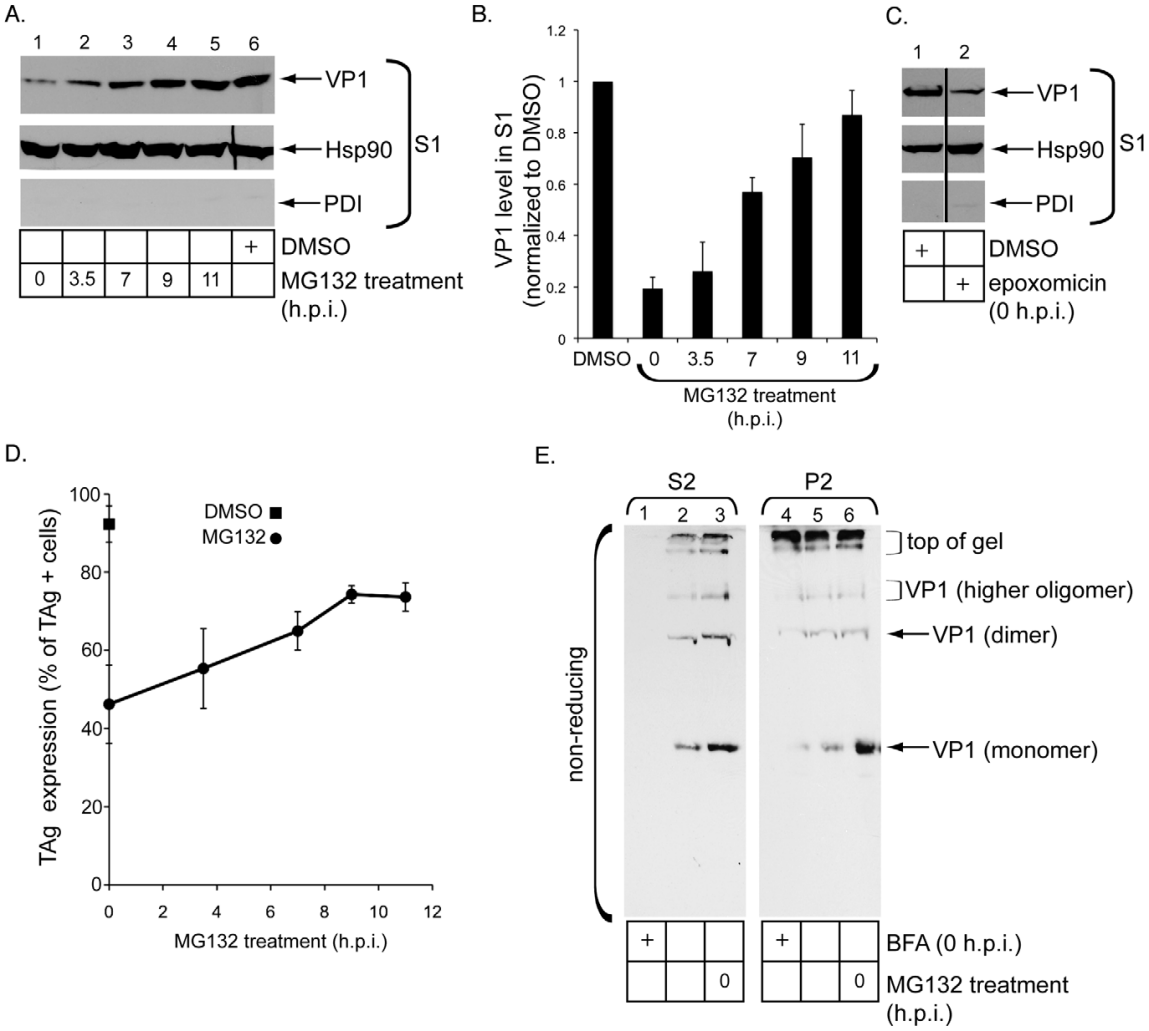
Release of SV40 into the cytosol depends on the host proteasome. (A) MG132 and DMSO were added to CV-1 cells at the indicated post-infection time points and at 0 h.p.i., respectively. Cells were harvested at 12 h.p.i. and subjected to the ER-to-cytosol membrane penetration assay. S1 was analyzed. (B) The VP1 band intensities in A were quantified with ImageJ (NIH). Data represent the mean +/− SD of at least 3 independent experiments. (C) As in A except where indicated, cells were treated with epoxomicin or DMSO at 0 h.p.i. (D) Cells were infected with SV40 and treated with MG132 or DMSO as in A, and analyzed as in Figure 1B. In a field of view, 320/359 cells scored TAg-positive in DMSO-treated cells. In MG132-treated cells, 114/205 cells scored TAg-positive at 0 h.p.i., 111/188 cells at 3.5 h.p.i., 155/221 cells at 7 h.p.i., 193/279 cells at 9 h.p.i., and 250/333 cells at 11 h.p.i. (E) Cells treated with or without BFA or MG132 were infected with SV40 for 12 hrs, processed to obtain the S2 and P2, and the sample subjected to non-reducing SDS-PAGE followed by immunoblotting against VP1.

What might be the driving force that discharges SV40 into the cytosol from the ER membrane? The proteasome has been shown to extract some misfolded proteins from the ER membrane into the cytosol [24], [25]. As proteasome inhibition decreased SV40 infection [12], we tested the proteasome's role in cytosol release of SV40 by using MG132, a proteasome inhibitor. When DMSO or MG132 was added simultaneously with SV40 to cells for 12 hrs, VP1 in S1 decreased in cells treated with MG132 when compared to DMSO (Figure 7A, top panel, compare lane 6 to 1; quantified in Figure 7B). The VP1 level in S1 was restored to a similar level as the DMSO-treated cells when MG132 was added increasingly later after incubation of cells with SV40 (Figure 7A, top panel, compare lane 6 to lanes 2–5; quantified in Figure 7B). The time range when proteasome inhibition no longer affects virus arrival to the cytosol (i.e. approximately 9–11 h.p.i.) occurs slightly after arrival of SV40 to the cytosol (i.e. approximately 8 h.p.i.). Addition of epoxomicin, a more specific proteasome inhibitor, to cells also decreased the S1 VP1 level at 12 h.p.i. (Figure 7C, top panel, compare lane 2 to 1), consistent with the MG132 effects. These findings indicate that the proteasome plays an important function in promoting virus release into the cytosol.

MG132 decreased SV40 infection when this drug was added simultaneously with SV40 to cells (Figure 7D, 0 h.p.i., compare square to circle), similar to a previous finding [12]. The infection level was restored partially if MG132 was added 9 or 11 h.p.i. (Figure 7D, circles), consistent with restoration of the S1 VP1 level when this drug was added at the same time points post-infection (Figure 7A and 7B). The correlation between the time-dependent effects of MG132 on viral infection and release into the cytosol underscores the proteasome's role in controlling SV40's ER-to-cytosol transport.

As inhibiting the proteasome prevents SV40 release into the cytosol, we hypothesized that such perturbation should concomitantly cause an increase in ER-localized virus. To assess the ER-localized SV40 level, we measured formation of viral disulfide bonded intermediates in the ER. Cells were incubated with SV40 for 12 hrs and subjected to the semi-permeabilized assay. The resulting P1 was used to generate S2 and P2. These fractions were subjected to non-reducing SDS-PAGE followed by immunoblotting with VP1-specific antibodies. We detected formation of VP1 monomer, dimer, and a low level of the higher oligomer in the S2 (Figure 7E, left panel, lane 2). BFA added at infection blocked the generation of these products (Figure 7E, left panel, compare lane 2 to 1), consistent with results observed in a WCE sample (Figure 1A). When cells were incubated simultaneously with MG132 and SV40, the VP1 monomer, dimer, and higher oligomer levels in the S2 increased when compared to control cells (Figure 7E, left panel, compare lane 3 to 2). Similarly, proteasome inhibition also increased VP1 monomer in P2 when analyzed by a non-reducing gel (Figure 7E, right panel, compare lane 6 to 5). Thus blocking the proteasome activity caused a build-up of virus in the ER lumen and those that remained attached to GM1 on the ER membrane. These findings further demonstrate a role of the proteasome in controlling exit of SV40 to the cytosol.

## Discussion

**Figure 8:**
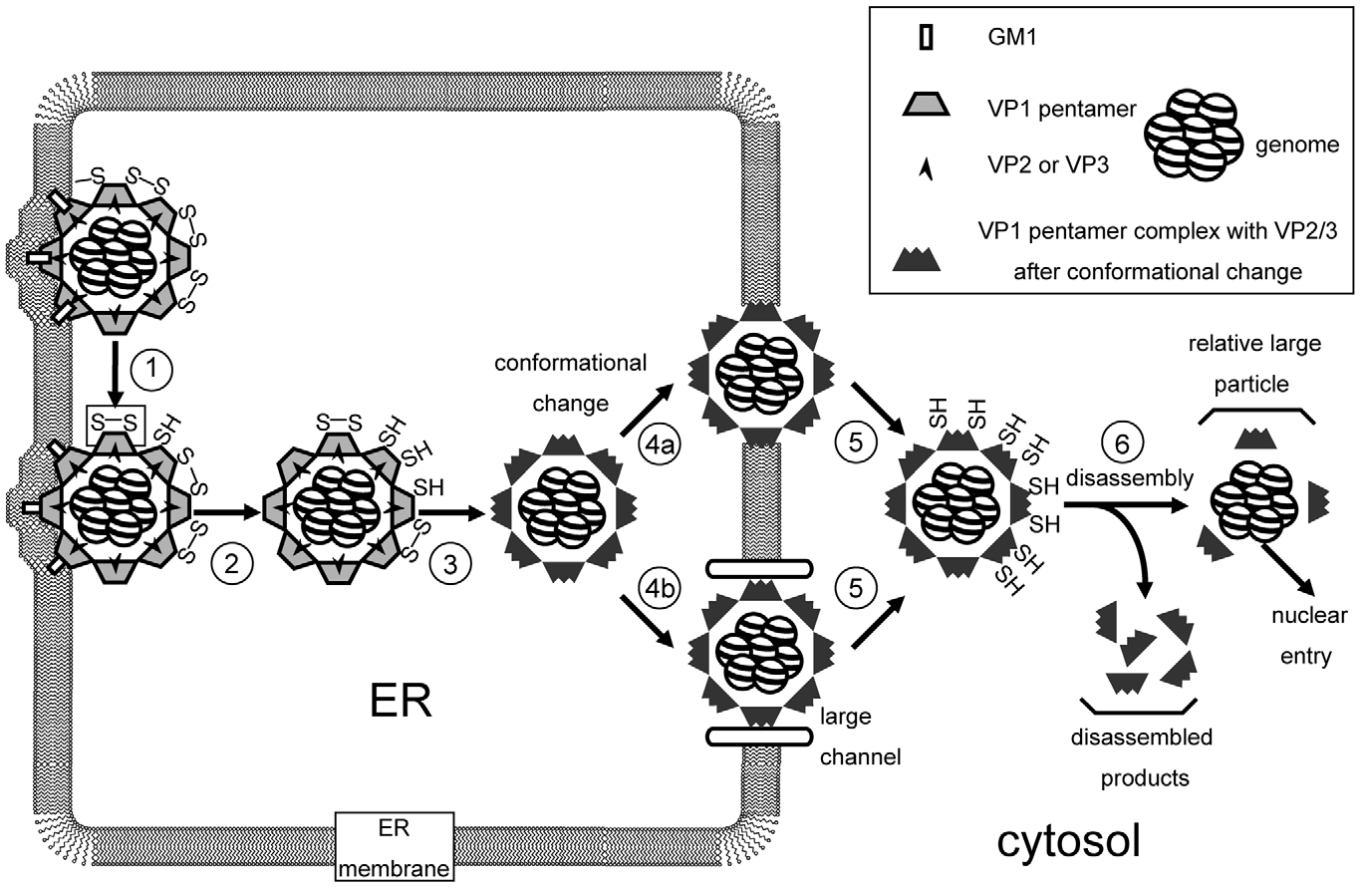
Model of SV40 penetration across the ER membrane (See text for discussion).

How non-enveloped viruses penetrate biological membranes is understood poorly [2]. Here we established a cell-based assay to examine ER-to-cytosol membrane transport of the non-enveloped SV40. Our findings drew four major conclusions, depicted in Figure 8. First, upon ER arrival, SV40 attached to GM1 on the ER membrane is released into the ER lumen, and undergoes sequential disulfide bond disruption to reach the cytosol (steps 1 and 2). Disulfide bond disruption triggers conformational changes that prime the virus for membrane penetration (step 3). This step may involve VP2 and VP3 exposure. Second, despite ER remodeling events, a large and intact viral particle penetrates the ER membrane to reach the cytosol, potentially through either the lipid bilayer (step 4a) or a sizeable protein-channel (step 4b). Third, viral VP3 and potentially the genome, as well as the host proteasome, regulate SV40 release into the cytosol (step 5). And fourth, SV40 disassembles in the cytosol (step 6). We will discuss these points separately.

### SV40 in the ER

Using a semi-permeabilized system, we found that SV40 is released into the ER lumen upon ER arrival, presumably by detaching from GM1. What might be the driving force for this reaction? ER factors may induce physical changes to VP1 that decreases its affinity for GM1. Alternatively, when GM1 reaches the ER, it may partition into the ER bilayer, thereby reducing SV40's affinity for the membrane.

In the ER, disruption of SV40's disulfide bonds by the PDI family members ERp57 and PDI imparts conformational changes on the viral particle, priming it for membrane penetration [12]. Using non-reducing SDS-PAGE, our analyses dissected this reaction into several steps. First, when SV40 attached to GM1 reaches the ER, its disulfide bonds are disrupted, generating VP1 monomer and dimer. Next, when the virus is released into the ER lumen, monomer and dimer, as well as a higher oligomer (which could be an intermediate for the dimer and monomer) continues to form. Because only a large viral particle was detected in the ER using non-SDS methods, disulfide bond disruption is not sufficient to generate VP1 monomer and dimer; the ER-localized SV40 likely represents VP1 pentamers that remain attached to the core viral particle via non-covalent interactions.

Finally, when the virus is discharged into the cytosol, all the intermediate species undergo a thorough disruption of the disulfide bonds to produce only VP1 monomer. During these steps, SV40's interchain Cys9-Cys9 and Cys104-Cys104 disulfide bonds [4], [12] are likely disrupted. As a species resembling VP1 pentamer (but not monomer) is detected in the cytosol using non-SDS methods, the monomer must be held together non-covalently. The sequential manner by which SV40's disulfide bonds are disrupted as it moves from the ER into the cytosol reveals the coordinated manner by which the host dismantles the virus.

### A large and intact SV40 penetrates the ER membrane

Using four independent biochemical approaches, our results unambiguously established that the conformations of the ER- and cytosol-localized viral particles are different. Specifically, we demonstrate that ER-localized SV40 is large and intact, and contains VP2, VP3, and the genome. No small viral particles were detected in the ER. In contrast, both large and small viral particles are present in the cytosol. These particles display weak VP1-VP2/VP3 binding. Furthermore, our EM analyses detected large 50 nm viral particles in the cytosol, although they appear to be heterogeneous in structure. The simplest interpretation of these data is that a large and intact viral particle in the ER penetrates the ER membrane to reach the cytosol where it disassembles.

Another potential explanation is that the ER-localized large particle disassembles to small particles that then discharge rapidly to the cytosol where they re-assemble into a large particle. However, this complex scenario is unlikely because it would require an unprecedented efficiency in removing all the small particles from the ER to the cytosol to preclude their detection in the ER. Moreover, it is also inconsistent with the established SV40 assembly process in which the nucleus but not the cytosol supports large virion assembly [20]. In our system, we further demonstrate that the cytosol does not provide an environment conducive for large particle assembly.

While a precise measurement of the large membrane penetrating species is not available, sucrose gradient analyses indicate that its size is similar to the native 50 nm SV40 virion. This proposed size raises the question of whether the virus crosses a protein-conducting channel or the ER lipid bilayer. A previous study implicated a role of Derlin-1, a component of an ER membrane complex used during ERAD [26], in SV40 infection [12]. Should Derlin-1 function as a channel, massive Derlin-1 oligomerization is required to accommodate viral transport. That biological membranes can support transport of large complexes is not without precedent, as a 9 nm gold particle decorated with the peroxisome-targeting signal can be transported into the peroxisome interior [27].

An alternative to the protein channel-based mechanism is a lipid-based strategy. Our in vitro findings on mPy provide insight into how this process may occur. The PDI family member ERp29 untangles the VP1 C-terminal arm of mPy in a reaction that requires reduction of the virus disulfide bonds and removal of the virus-bound calciums [28]. VP2 and possibly VP3 are then exposed, generating a hydrophobic viral particle that binds, integrates, and perforates the ER membrane [28], [29]. These reactions initiate mPy's penetration across the ER lipid bilayer. Interestingly, a different version of the lipid-based model was hypothesized [30]. In this model, a pore in the ER membrane created when a lipid droplet leaves the membrane enables SV40/mPy to gain access to the cytosol. No experiments have validated this idea thus far.

### Role of viral and host components in regulating ER membrane penetration

While SV40 VP2 and VP3 have been implicated in nuclear entry [23], our findings demonstrate that at least VP3 plays a role in SV40's ER-to-cytosol transport; our results cannot distinguish any function of VP2 in this process. In vitro, SV40 VP2 and VP3 can integrate into the ER membrane [31]. Integration of these proteins into the ER membrane may create a pore through which the viral genome is injected [31]. Alternatively, VP2 and VP3 may act as lytic factors [32], perforating the ER membrane to allow passage of a subviral particle. As the ER and nuclear membranes are continuous, a subviral particle could bypass the cytosol and reach the nucleus directly after penetrating the ER membrane. However, the findings that cytosol arrival is required for SV40 infection [33], that interaction between VP3's nuclear localization signal and importins is necessary for nuclear entry [34], and that ER machineries dedicated to ERAD are crucial for infection [12], point to the ER-to-cytosol transport pathway as the dominant infectious route. As SV40's genome stabilizes its overall viral architecture [12], its absence likely destabilizes the virus structure. This could in turn lead to incorrect conformational changes that perturb ER-to-cytosol transport.

The host proteasome also plays a pivotal function in controlling SV40's ER-to-cytosol transport. Since the proteasome extracts some misfolded ER proteins to the cytosol [24], [25], it may also discharge SV40 into the cytosol. Establishing a cell-free reconstituted system will reveal if the proteasome plays a direct role in viral release.

Our data also suggest that VP2 controls SV40 sorting to the ER from the cell surface. In addition, VP3 may also be involved in this process, should an SV40 mutant virus lacking VP3 reach the ER inefficiently after 6 h.p.i.. Further experiments are required to clarify how VP2 regulates ER sorting, and whether VP3 plays any role.

### Cytosol disassembly

Our analyses demonstrated that SV40 disassembles in the cytosol. The starting substrate for this reaction is a large particle that reaches the cytosol from the ER. Indeed, large particles approximating 50 nm were detected in the cytosol by EM. Their heterogeneous nature may reflect the various disassembly intermediates. Of particular interest is the viral intermediate containing a doughnut-shaped pore in the middle of its structure. This species might represent a viral particle in which a 5-coordinated VP1 pentamer is released to generate a pore. Release of the 5-coordinated VP1 pentamer from intact SV40 in vitro was previously hypothesized to be involved in ER-to-cytosol transport [12].

Our biochemical results also show that the large cytosol-localized virus disassembles to generate small particles approximating the size of a pentamer and lacks the genome. This disassembly reaction may be aided by the low calcium concentration in the cytosol which would promote loss of calcium ions from the cytosol-localized virus, thereby further destabilizing VP1 capsomer interaction. The remaining core particle (relatively large particle in Figure 8), which harbors the genome, is likely targeted to the nucleus to cause infection. As the cytosol-localized viral intermediates observed by EM are large, they are unlikely the species that enter the nucleus. Because previous studies showed that Hsp70 uncoats mPy in vitro [35] and binds to SV40 in cells [36], this cytosolic chaperone may convert the large SV40 particle to the small particle in our assay. Whether cytosolic disassembly is coupled to nuclear entry is unknown, and unraveling it will provide insight into another critical step in SV40's infection pathway.

## Materials and Methods

### Materials

Polyclonal antibodies against Hsp90 and PDI were purchased from Santa Cruz Biotechnology, monoclonal antibodies against PDI from Abcam, large T antigen from Santa Cruz Biotechnology, MG132 and epoxomicin from EMD chemicals, BFA from Epicenter, proteinase K and monoclonal antibodies against HA from Roche, and TCEP from Thermo Scientific. All other reagents were from Sigma. The pUCSV40 encoding SV40 genome and polyclonal antibodies against VP1 were generous gifts from Dr. H. Handa (Tokyo Institute of Technology), polyclonal antibodies against VP3 from Dr. H. Kasamatsu (University of California, Los Angeles) and monoclonal antibodies against VP1 from Dr. W. Scott (University of Miami).

### ER-to-cytosol membrane penetration assay

CV-1 cells were incubated with SV40 (m.o.i. = 3–50) at 4°C, washed, and incubated at 37°C. At indicated time points, cells were trypsinized (scraped off for the mutant viruses), permeabilized with HN buffer (50 mM Hepes, pH 7.5, 150 mM NaCl, and protease inhibitors) containing 0.1% digitonin on ice for 10 min, and centrifuged at 16,100 *g* for 10 min. The resulting supernatant is referred as S1. The pellet was resuspended in SDS sample buffer and is referred as P1. Where indicated, P1 was incubated in HN buffer containing 1% Triton X-100 on ice for 10 min and centrifuged at 16,100 *g* for 10 min. This second supernatant is referred as S2. The Triton X-100-insoluble pellet was resuspended in SDS sample buffer and is referred to as P2. For non-reducing SDS-PAGE, NEM (10 mM) was added to all buffers.

### Immunoprecipitation

SV40 monoclonal antibodies (CC10 and BC11) or an HA monoclonal antibody were added to S1 and S2 and incubated on ice for 3 hrs. Protein G-Dynabeads (Invitrogen) were used to capture the antibody-virus complex. The beads were isolated using a magnet stand (Dynal), washed with a high salt buffer (50 mM Hepes, pH 7.5, 500 mM NaCl, 1% Triton X-100), and the bound proteins eluted with an acidic buffer (50 mM glycine, pH 2.8).

### Infection

CV-1 cells were incubated with the indicated viruses at 4°C for 2 hrs. The cells were washed and incubated at 37°C. 24 h.p.i., cells were fixed with 1% paraformaldehyde, treated with 0.2% Triton X-100, and incubated in 3% milk. The cells were stained with a mouse monoclonal SV40 large T antigen antibody, followed by Alexa Fluor-488-conjugated secondary antibody (Invitrogen). In each experiment, approximately 1,000 cells were counted to assess the extent of large T antigen expression.

### Gel filtration

S1 and S2 were loaded onto a Bio-Sil 600 gel filtration column (Bio-Rad) and separated with HN buffer. Forty fractions (0.5 ml each) were collected and 0.1 ml of fractions 9-30 was separated by SDS-PAGE, followed by immunoblotting with VP1 monoclonal antibodies.

### Continuous sucrose gradient

S1 and S2 were loaded onto a 0.5 ml preformed 20–40% sucrose gradient and centrifuged at 49,500 rpm for 50 min at 4°C in an SW 55Ti rotor. After centrifugation, 10 fractions were collected from the top.

### Sucrose cushion sedimentation

S1, S2, and WT SV40 were layered over a 20% sucrose solution, centrifuged, and the sedimented material and material near the top of the cushion were subjected to immunoblotting.

### SV40 transfection

Cells were transfected (Lipofectamine 2000, Invitrogen) with the SV40 genome for 48 hrs, harvested, and subjected to the ER-to-cytosol assay to generate S1 and P1. P1 was freeze-thawed to extract virus from the nucleus. Both fractions were analyzed by sucrose gradient sedimentation.

### Preparation of WT and mutant SV40

WT and SV40 mutants were purified using the OptiPrep gradient system, except SV40 (-genome) was purified by CsCl gradient. Briefly, SV40-infected or viral genome-transfected CV-1 cells were lysed in a buffer containing 50 mM Hepes (pH 7.5), 150 mM NaCl, and 0.5% Brij58 on ice for 30 min and centrifuged at 16,100 g for 10 min. The supernatant was loaded onto a discontinuous 20 and 40% OptiPrep gradient and centrifuged at 49,500 rpm for 2 hrs at 4°C in an SW 55Ti rotor. A viral particle fraction between 20% and 40% OptiPrep was collected with a needle. For separation of virion and empty particle, supernatant was loaded onto a 1.516, 1.443, 1.37, 1.296, 1.222, and 1.148 g/ml discontinuous CsCl gradient (1 ml each) and centrifuged at 35,000 rpm for 3 hrs at 4°C in an SW 41Ti rotor. Fractions corresponding to virion and empty particle were collected. Each fraction was transferred into a 5×41-mm open-top tube (Beckman) and centrifuged at 49,500 rpm for 12 hrs at 4°C in an SW 55Ti rotor. A fraction corresponding to virion or empty particle was collected.

### Biotinylated SV40

Purified SV40 was labeled with EZ-Link Sulfo-NHS-LC-Biotin (Thermo) according to the manufacturer's protocol.

### siRNA knock-down

33 nM ERp57-specific (5′-UGAAGGUGGCCGUGAAUUATT-3′) (Invitrogen) or control (Ambion) siRNAs were transfected into CV-1 cells using the Lipofectamine 2000 system according to the manufacturer's protocol. At 36 hrs post-infection, cells were infected with SV40 at m.o.i. = 5 and subjected to the ER-to-cytosol membrane penetration assay.

### Viral genome detection by PCR

Samples from S1, immunoprecipitation, or sucrose gradient fractions were incubated in 10 mM Tris-HCl (pH 8.5) containing 0.2 mg/ml proteinase K. After proteinase K was heat-inactivated, the samples were subjected to a PCR reaction using a set of primers (GCAGTAGCAATCAA CCCACA [forward] and CTGACTTTGGAGGCTTCTGG [reverse]).

### ER co-localization

CV-1 cells plated on 18 mm glass plates were washed with DMEM, chilled at 4°C, and incubated with SV40 (m.o.i. = 1) at 4°C for 1 hr. Cells were washed extensively to remove unbound viruses, incubated in DMEM at 37°C for 10 hrs, fixed with 1% paraformaldehyde, incubated with a mouse monoclonal SV40 VP1 and rabbit polyclonal PDI antibody, followed by an Alexa Fluor 594 and Rhodamine conjugated secondary antibodies. Images were taken with an Olympus FV-500 confocal microscopy equipped with 100x objective. The ER images derived from the PDI signal were subjected to the FFT Bandpass Filter embedded in Image J (NIH) as described previously [36].

### Trypsin digestion analysis

4-fold more S1 at 4 h.p.i. was used to ensure that the VP1 level is similar between S1 at 4 and 12 h.p.i. The samples were incubated with 30 or 100 µg/ml trypsin for 1 hr on ice and the reaction was stopped by the addition of 1 mM TLCK for 10 min on ice. The samples were separated by SDS-PAGE followed by immunoblotting with SV40 VP1 monoclonal antibodies.

### OptiPrep flotation assay

S1 or purified SV40 was mixed with the same amount of 60% OptiPrep solution. 100 µl of the mixed sample was placed at the bottom of a Beckman centrifuge tube (7×20 mm), and 100 µl of 20% OptiPrep was loaded onto the sample. The tube was centrifuged in a Beckman TLA100 rotor for 1 hr at 100,000 rpm. Fractions were collected from the top (20 µl each), separated by SDS-PAGE, and immunoblotted with SV40 VP1 monoclonal antibodies.

### CT intoxication

CV-1 cells were intoxicated with 30 nM CT and subjected to semi-permeabilization with 0.1% digitonin as described in the ER-to-cytosol membrane penetration assay. S1 and P1 fractions were analyzed by SDS-PAGE followed by immunoblotting with CTA, PDI, and Hsp90 antibodies.

### Isolation of CT in vesicles

Cells were washed with DMEM, chilled at 4°C in 10 ml of DMEM for 20 min, and incubated with 30 nM CT at 4°C for 2 hrs. Cells were then washed with cold PBS to remove unbound CT and incubated in 10 ml of DMEM at 37°C to allow entry. At the indicated time points, cells were washed with cold PBS, scraped off the plate in 1 ml of PBS containing 10 mM NEM, and collected in a microcentrifuge tube. S1, prepared as described above, was incubated with or without 2% SDS at 25°C for 10 min. The samples were subjected to high-speed centrifugation in a Beckman TLA100 rotor for 30 min at 100,000 g. The resulting supernatant and pellet fractions after high-speed spin, and the original S1, were analyzed by SDS-PAGE followed by immunoblotting with polyclonal CTB antibodies.

### Negaive stain electron microscopy (EM)

S1, prepared from cells (7.5×10^6^ cells) infected with SV40 for 12 hrs, was incubated with 1% Triton X-100 to solubilize any membrane material, centrifuged in a Beckman TLA100 rotor for 30 min at 100,000 g to concentrate the virus, the resulting pellet resuspended in 100 µl of HN buffer, and subjected to immunoprecipitation as described above. The virus-antibody-bead complex was captured by a magnet stand (Dynal) and washed with HN buffer containing 1% Triton X-100. The magnetic beads were resupended in 20 µl of HN buffer. For negative staining, 5 µl of each sample containing magnetic beads were absorbed onto a grow-discharged copper grid (Electron Microscopy Sciences) and stained with 1% uranyl acetate. The samples were observed using a Philips CM-100 at 80 kV.

## Supporting Information

Figure S1 Additional characterization of the ER-to-cytosol penetration assay. (A) SV40-infected cells were treated with or without BFA 4 h.p.i., and infection continued for 8 more hrs. WCE was prepared and analyzed by non-reducing and reducing SDS-PAGE, and immunoblotted with an antibody against VP1. (B) Cells plated on 18 mm glass plates were washed with DMEM, chilled at 4°C, and incubated with SV40 (m.o.i. = 1) at 4°C for 1 hr. Cells were then washed extensively to remove unbound viruses, incubated in DMEM at 37°C for 10 hrs, fixed with 1% paraformaldehyde, incubated with a mouse monoclonal SV40 VP1 and rabbit polyclonal PDI antibody, followed by an Alexa Fluor 594 and Rhodamine conjugated secondary antibodies. Images were taken with an Olympus FV-500 confocal microscopy equipped with 100x objective. White arrow head represents SV40 particle that co-localized completely with the ER, while white arrow represents those virus that either did not co-localize with the ER or co-localized with the ER partially. (C) Cells were subjected to the semi-permeabilized fractionation method to generate S1 and P1. These fractions were subjected to SDS-PAGE followed by immunoblotting using antibodies against Hsp90 and actin. (D) Cells treated with or without BFA at intoxication were incubated with CT for 90 min. Cells were then subjected to the semi-permeabilized assay to generate S1 and P1. These fractions were subjected to SDS-PAGE followed by immunoblotting against the indicated antibodies. (E) Cells were incubated with SV40 for the indicated amount of time and processed according to the ER-to-cytosol transport assay to generate S1. Samples were subjected to SDS-PAGE and immunoblotted with the indicated antibodies. (F) Cells incubated with CTB for 5 or 90 min were processed according to the semi-permeabilized assay to generate S1. S1 was treated with or without SDS and centrifuged at high-speed (100,000 g) to produce a supernatant (sn) and pellet fraction. S1, sn, and pellet fractions were subjected to SDS-PAGE followed by immunoblotting using an antibody against CTB. (G) S1 was generated from cells infected with SV40 for 4 or 12 hrs. S1 were treated with or without the indicated trypsin concentration and subjected to SDS-PAGE followed by immnoblotting with an antibody against VP1. 4-fold more S1 derived from cells infected with SV40 for 4 hrs when compared to 12 hrs were used. (H) S1 samples in G, as well as WT SV40, were subjected to OptiPrep gradient flotation. Individual fractions were subjected to SDS-PAGE followed by immunoblotting against VP1. (I) Cells infected with the indicated SV40 concentration for 12 hrs were processed according to the semi-permeabilized assay to generate S1 and P1. These fractions were subjected to SDS-PAGE followed by immunoblotting using the indicated antibodies. (graph) Infection studies using the different SV40 concentrations were performed according to Figure 1B. In a field of view, 156/372 cells scored TAg-positive at m.o.i. = 3, 167/297 cells at m.o.i. = 5, 314/427 cells at m.o.i. = 10, 355/415 cells at m.o.i. = 30, and 350/371 at m.o.i. = 50. (J) Cells treated with or without DTT (1 mM) were infected with SV40 for 12 hrs and processed according to the semi-permeabilized assay to generate S1 and P1. These fractions were subjected to SDS-PAGE followed by immunoblotting using the indicated antibodies. (graph) Infection studies wee performed according to Figure 1B. In a field of view, 384/456 cells scored TAg-positive in control cells, and 111/333 cells TAg-positive in DTT-treated cells. (K) Cells treated with or without BFA were infected with SV40 labeled with biotin for 12 hrs. The cells were processed according to the semi-permeabilized assay to generate S1 and P1. These fractions were subjected to SDS-PAGE followed by immunoblotting using the indicated antibodies.
(TIF)

## References

[1] Chandran K, Nibert ML (2003). Animal cell invasion by a large nonenveloped virus: reovirus delivers the goods.. Trends Microbiol.

[2] Tsai B (2007). Penetration of nonenveloped viruses into the cytoplasm.. Annu Rev Cell Dev Biol.

[3] Liddington RC, Yan Y, Moulai J, Sahli R, Benjamin TL (1991). Structure of simian virus 40 at 3.8-A resolution.. Nature.

[4] Stehle T, Gamblin SJ, Yan Y, Harrison SC (1996). The structure of simian virus 40 refined at 3.1 A resolution.. Structure.

[5] Chen XS, Stehle T, Harrison SC (1998). Interaction of polyomavirus internal protein VP2 with the major capsid protein VP1 and implications for participation of VP2 in viral entry.. Embo J.

[6] Tsai B, Gilbert JM, Stehle T, Lencer W, Benjamin TL (2003). Gangliosides are receptors for murine polyoma virus and SV40.. Embo J.

[7] Ewers H, Romer W, Smith AE, Bacia K, Dmitrieff S (2010). GM1 structure determines SV40-induced membrane invagination and infection.. Nat Cell Biol.

[8] Pelkmans L, Kartenbeck J, Helenius A (2001). Caveolar endocytosis of simian virus 40 reveals a new two-step vesicular-transport pathway to the ER.. Nat Cell Biol.

[9] Mercer J, Schelhaas M, Helenius A (2010). Virus entry by endocytosis.. Annu Rev Biochem.

[10] Qian M, Tsai B (2010). Lipids and proteins act in opposing manners to regulate polyomavirus infection.. J Virol.

[11] Norkin LC, Anderson HA, Wolfrom SA, Oppenheim A (2002). Caveolar endocytosis of simian virus 40 is followed by brefeldin A-sensitive transport to the endoplasmic reticulum, where the virus disassembles.. J Virol.

[12] Schelhaas M, Malmstrom J, Pelkmans L, Haugstetter J, Ellgaard L (2007). Simian Virus 40 depends on ER protein folding and quality control factors for entry into host cells.. Cell.

[13] Damm EM, Pelkmans L, Kartenbeck J, Mezzacasa A, Kurzchalia T (2005). Clathrin- and caveolin-1-independent endocytosis: entry of simian virus 40 into cells devoid of caveolae.. J Cell Biol.

[14] Qian M, Cai D, Verhey KJ, Tsai B (2009). A lipid receptor sorts polyomavirus from the endolysosome to the endoplasmic reticulum to cause infection.. PLoS Pathog.

[15] Forster ML, Sivick K, Park YN, Arvan P, Lencer WI (2006). Protein disulfide isomerase-like proteins play opposing roles during retrotranslocation.. J Cell Biol.

[16] Bernardi KM, Forster ML, Lencer WI, Tsai B (2008). Derlin-1 facilitates the retro-translocation of cholera toxin.. Mol Biol Cell.

[17] Lencer WI, Tsai B (2003). The intracellular voyage of cholera toxin: going retro.. Trends Biochem Sci.

[18] Resnick J, Shenk T (1986). Simian virus 40 agnoprotein facilitates normal nuclear location of the major capsid polypeptide and cell-to-cell spread of virus.. J Virol.

[19] Lin W, Hata T, Kasamatsu H (1984). Subcellular distribution of viral structural proteins during simian virus 40 infection.. J Virol.

[20] Brown DA, London E (1998). Functions of lipid rafts in biological membranes.. Annu Rev Cell Dev Biol.

[21] Kartenbeck J, Stukenbrok H, Helenius A (1989). Endocytosis of simian virus 40 into the endoplasmic reticulum.. J Cell Biol.

[22] Babe LM, Brew K, Matsuura SE, Scott WA (1989). Epitopes on the major capsid protein of simian virus 40.. J Biol Chem.

[23] Nakanishi A, Itoh N, Li PP, Handa H, Liddington RC (2007). Minor capsid proteins of simian virus 40 are dispensable for nucleocapsid assembly and cell entry but are required for nuclear entry of the viral genome.. J Virol.

[24] Lee RJ, Liu CW, Harty C, McCracken AA, Latterich M (2004). Uncoupling retro-translocation and degradation in the ER-associated degradation of a soluble protein.. Embo J.

[25] Mayer TU, Braun T, Jentsch S (1998). Role of the proteasome in membrane extraction of a short-lived ER-transmembrane protein.. Embo J.

[26] Vembar SS, Brodsky JL (2008). One step at a time: endoplasmic reticulum-associated degradation.. Nat Rev Mol Cell Biol.

[27] Walton PA, Hill PE, Subramani S (1995). Import of stably folded proteins into peroxisomes.. Mol Biol Cell.

[28] Magnuson B, Rainey EK, Benjamin T, Baryshev M, Mkrtchian S (2005). ERp29 triggers a conformational change in polyomavirus to stimulate membrane binding.. Mol Cell.

[29] Rainey-Barger EK, Magnuson B, Tsai B (2007). A chaperone-activated nonenveloped virus perforates the physiologically relevant endoplasmic reticulum membrane.. J Virol.

[30] Ploegh HL (2007). A lipid-based model for the creation of an escape hatch from the endoplasmic reticulum.. Nature.

[31] Daniels R, Rusan NM, Wadsworth P, Hebert DN (2006). SV40 VP2 and VP3 insertion into ER membranes is controlled by the capsid protein VP1: implications for DNA translocation out of the ER.. Mol Cell.

[32] Daniels R, Rusan NM, Wilbuer AK, Norkin LC, Wadsworth P (2006). Simian virus 40 late proteins possess lytic properties that render them capable of permeabilizing cellular membranes.. J Virol.

[33] Nakanishi A, Clever J, Yamada M, Li PP, Kasamatsu H (1996). Association with capsid proteins promotes nuclear targeting of simian virus 40 DNA.. Proc Natl Acad Sci U S A.

[34] Nakanishi A, Shum D, Morioka H, Otsuka E, Kasamatsu H (2002). Interaction of the Vp3 nuclear localization signal with the importin alpha 2/beta heterodimer directs nuclear entry of infecting simian virus 40.. J Virol.

[35] Chromy LR, Oltman A, Estes PA, Garcea RL (2006). Chaperone-mediated in vitro disassembly of polyoma- and papillomaviruses.. J Virol.

[36] Li PP, Itoh N, Watanabe M, Shi Y, Liu P (2009). Association of simian virus 40 vp1 with 70-kilodalton heat shock proteins and viral tumor antigens.. J Virol.

