# Supplementary figures and images for: A Large and Intact Viral Particle Penetrates the Endoplasmic Reticulum Membrane to Reach the Cytosol
Source: PLoS Pathog. 2011 May 12;7(5):e1002037. doi: 10.1371/journal.ppat.1002037 (PMC3093372; doi:10.1371/journal.ppat.1002037)

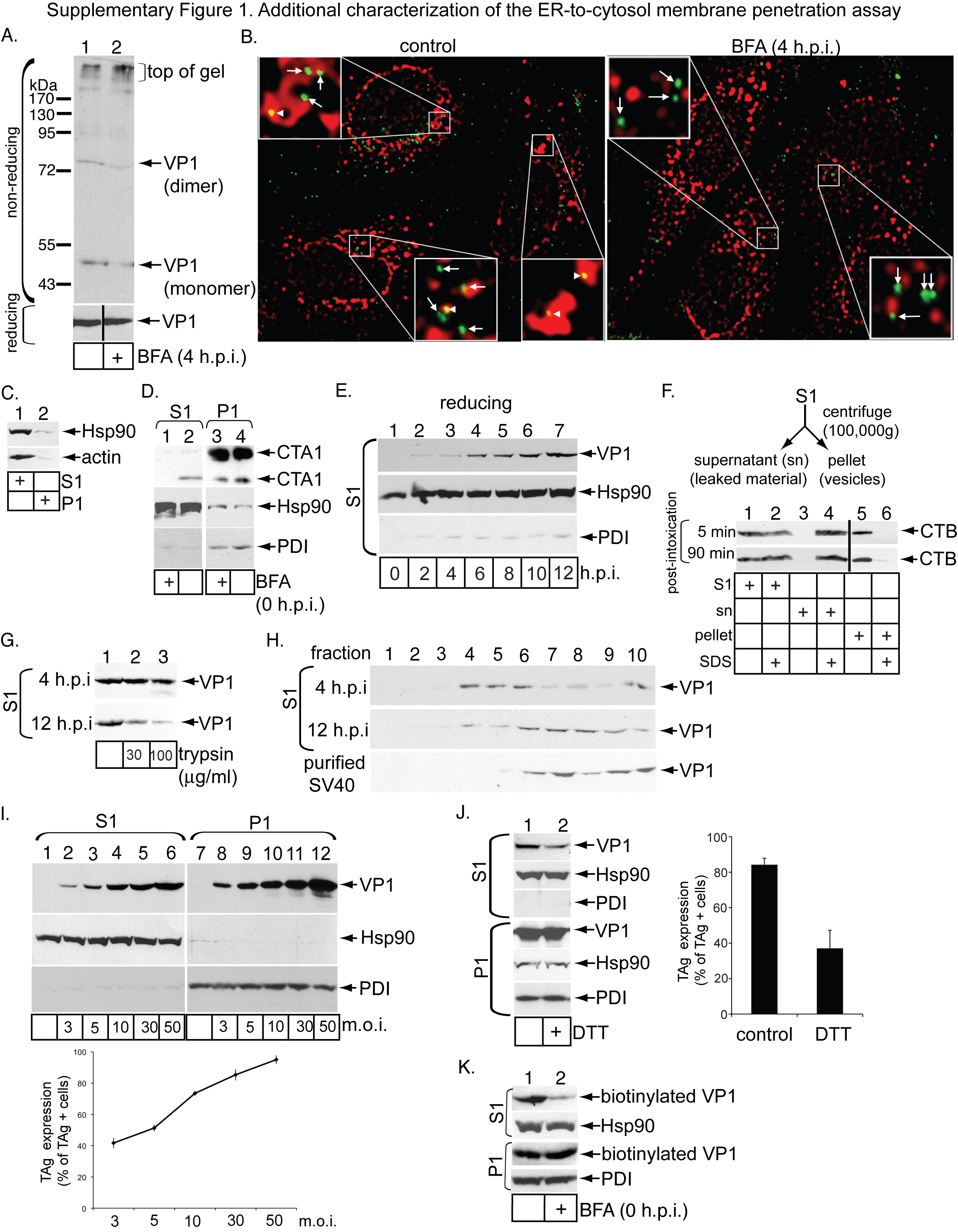

Supplement: Figure S1 — Additional characterization of the ER-to-cytosol penetration assay. (A) SV40-infected cells were treated with or without BFA 4 h.p.i., and infection continued for 8 more hrs. WCE was prepared and analyzed by non-reducing and reducing SDS-PAGE, and immunoblotted with an antibody against VP1. (B) Cells plated on 18 mm glass plates were washed with DMEM, chilled at 4°C, and incubated with SV40 (m.o.i. = 1) at 4°C for 1 hr. Cells were then washed extensively to remove unbound viruses, incubated in DMEM at 37°C for 10 hrs, fixed with 1% paraformaldehyde, incubated with a mouse monoclonal SV40 VP1 and rabbit polyclonal PDI antibody, followed by an Alexa Fluor 594 and Rhodamine conjugated secondary antibodies. Images were taken with an Olympus FV-500 confocal microscopy equipped with 100x objective. White arrow head represents SV40 particle that co-localized completely with the ER, while white arrow represents those virus that either did not co-localize with the ER or co-localized with the ER partially. (C) Cells were subjected to the semi-permeabilized fractionation method to generate S1 and P1. These fractions were subjected to SDS-PAGE followed by immunoblotting using antibodies against Hsp90 and actin. (D) Cells treated with or without BFA at intoxication were incubated with CT for 90 min. Cells were then subjected to the semi-permeabilized assay to generate S1 and P1. These fractions were subjected to SDS-PAGE followed by immunoblotting against the indicated antibodies. (E) Cells were incubated with SV40 for the indicated amount of time and processed according to the ER-to-cytosol transport assay to generate S1. Samples were subjected to SDS-PAGE and immunoblotted with the indicated antibodies. (F) Cells incubated with CTB for 5 or 90 min were processed according to the semi-permeabilized assay to generate S1. S1 was treated with or without SDS and centrifuged at high-speed (100,000 g) to produce a supernatant (sn) and pellet fraction. S1, sn, and pellet fractions wer [file ppat.1002037.s001.tif]
